# Bispecific Nanosystems Enable Multieffector Immune Cell Retargeting for Hematologic Malignancy Therapy

**DOI:** 10.1002/advs.202509103

**Published:** 2025-08-11

**Authors:** Yefeng Shen, Xin Li, Jingnan Wu, Yuru Ma, Sven Borchmann, Zhenguo Cheng, Yaohe Wang, Yongliang Zhao, Jian Song, Boyu Luo, Xiuyun Liu, Yue Teng, Zhiyuan Shi

**Affiliations:** ^1^ School of Pharmaceutical Science and Technology Faculty of Medicine Tianjin University Tianjin 300072 China; ^2^ Department of Thoracic Surgery Beijing Friendship Hospital Capital Medical University Beijing 100050 China; ^3^ Department of Biomedical Engineering City University of Hong Kong Hong Kong 999077 China; ^4^ State Key Laboratory of Pathogen and Biosecurity Academy of Military Medical Sciences Beijing 100071 China; ^5^ Institute of Technical and Macromolecular Chemistry RWTH Aachen University 52074 Aachen Germany; ^6^ Department I of Internal Medicine University of Cologne 50937 Köln Germany; ^7^ National Centre for International Research in Cell and Gene Therapy Sino‐British Research Centre for Molecular Oncology State Key Laboratory of Esophageal Cancer Prevention & Treatment School of Basic Medical Sciences Academy of Medical Sciences Zhengzhou University Zhengzhou 450001 China; ^8^ Centre for Cancer Biomarkers & Biotherapeutics Barts Cancer Institute Queen Mary University of London London EC1M 6BQ UK; ^9^ Ningbo Dilato Materials Co., LTD 581 South Zhuangyu Road, Zhenhai District Ningbo 315200 China; ^10^ Institute of Cardiovascular Sciences Guangxi Academy of Medical Sciences Nanning 530021 China; ^11^ Neurocritical Care Medicine Innovation Center Ministry of Education Tianjin University Tianjin 300072 China

**Keywords:** B cell lymphoma, bispecific antibody, cancer immunotherapy, hollow silica nanoparticles, precision nanomedicine

## Abstract

B‐cell lymphomas are hematologic malignancies characterized by poor prognoses. Immunotherapy has revolutionized B‐cell lymphoma treatment by harnessing immune effector cells, but current therapeutic strategies face limitations: suboptimal pharmacokinetics of bispecific antibodies and high complexity and cost of chimeric antigen receptor T‐cell therapies. To address these challenges, a bispecific nanosystem (biHSNPs) is developed that exploits the multi‐functional customizability of silica nanoplatform to conjugate antibodies targeting cytotoxic T cells or natural killer cells, alongside effector antibodies specific to B‐cells. Four biHSNPs with different effector and target antibodies are synthesized. This bispecific nanosystem enables simultaneous binding to immune effector cells and B‐cell lymphoma antigens, facilitating the formation of artificial immunological synapses. These synapses promote immune effector cell activation, leading to the release of cytotoxic proteins, while concurrently suppressing tumor cell proliferation and enhancing T‐cell activation. In vivo, biHSNPs effectively suppress tumor growth and activate T cells in a xenograft mouse model, showcasing their potential in precision therapy. Moreover, biHSNPs successfully overcome tumor immune evasion through dual‐target signal blockade. Using a straightforward and scalable strategy, a bispecific nanosystem is constructed that not only addresses the limitations of current bispecific antibody therapies but also represents a promising approach for the treatment of hematological malignancies.

## Graphical abstract

B‐cell lymphomas are aggressive malignancies with limited treatment options. A bispecific nanosystem (biHSNPs) is developed, leveraging silica nanoplatforms to conjugate antibodies targeting immune effector cells and B‐cell antigens. biHSNPs form artificial immunological synapses, activating cytotoxic responses and suppressing tumor proliferation. This scalable approach overcomes limitations of current immunotherapies, offering a promising precision therapy for hematological malignancies.

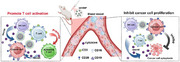

## 1 Introduction

B‐cell lymphomas, including Hodgkin lymphoma and most non‐Hodgkin lymphomas, are cancers arising from the abnormal growth and proliferation of B lymphocytes.^[^ ^1^ ^]^ They are classified as indolent (low‐grade) or aggressive (high‐grade) subtypes. Common tumor‐associated antigens, such as CD19 and CD20, serve as key therapeutic targets.^[^ ^1b^ ^]^ Current treatment options primarily include surgery, chemotherapy, and radiation, which remain the mainstays of therapy due to the limited applicability and effectiveness of immunotherapies.^[^ ^2^ ^]^ Early‐stage indolent lymphomas often achieve long‐term remission with radiation alone, while aggressive lymphomas are treated with chemotherapy, achieving cure rates of 70–90%. However, outcomes for patients with relapsed aggressive B‐cell lymphomas remain poor, with most succumbing to the disease.^[^ ^2^, ^3^ ^]^ Furthermore, conventional therapies, such as radiation and chemotherapy, carry significant risks of long‐term complications, including secondary cancers and organ damage, underscoring the urgent need for safer and more effective treatment strategies.^[^ ^4^ ^]^

Recent advancements in cancer immunotherapy have leveraged a deeper understanding of tumor‐immune system interactions,^[^ ^3^, ^5^ ^]^ driving the development of innovative therapies aimed at reactivating immune responses and redirecting immune cells to tumor sites.^[^ ^6^ ^]^ Currently, four major immunotherapeutic approaches are employed in the treatment of hematological cancers: immune checkpoint inhibitors,^[^ ^7^ ^]^ chimeric antigen receptor (CAR) T‐cells,^[^ ^6a,b^ ^]^ monoclonal antibodies,^[^ ^5^, ^8^ ^]^ and bispecific antibodies.^[^ ^8^, ^9^ ^]^ While these modalities have advanced the treatment landscape, each has limitations. Immune checkpoint inhibitors have achieved remarkable success in certain solid tumors but show limited efficacy in hematological cancers, with benefits restricted to select malignancies like Hodgkin lymphoma. CAR T‐cell therapies, which involve engineering T cells to express chimeric antigen receptors that recognize specific tumor antigens, have demonstrated efficacy in acute lymphoblastic leukemia and diffuse large B‐cell lymphoma (DLBCL).^[^ ^6^, ^10^ ^]^ However, their application is hindered by high cost, long manufacturing timelines, and severe side effects such as cytokine release syndrome. Monoclonal antibodies, such as Rituximab and Obinutuzumab, target tumor antigens like CD20 but may exhibit limited efficacy due to antigen escape.^[^ ^9^, ^11^ ^]^ Notably, the emerging bispecific antibodies, which can simultaneously target tumor antigens and immune effector cell receptors, represent a promising alternative. CD19 and CD20 are reliable therapeutic targets in B‐cell malignancies, while CD3 and CD16 provide potent immune activation pathways.^[^ ^12^ ^]^ For instance, CD19, expressed throughout B‐cell development, facilitates cell signaling and immune regulation,^[^ ^13^ ^]^ while CD20, predominantly found on mature B cells, plays a critical role in B‐cell proliferation and differentiation.^[^ ^14^ ^]^ Targeted therapies, including CD20‐directed monoclonal antibodies and CD19‐targeting CAR T‐cells, have significantly advanced the treatment landscape of B‐cell lymphomas. Nevertheless, conventional bispecific antibodies suffer from limitations such as short half‐life, necessitating continuous infusion (e.g., Blinatumomab), and cumbersome production processes.^[^ ^15^ ^]^ Thus, a novel therapeutic method is needed—one that integrates multiple immune targets, avoids the toxicity of traditional therapies, and offers effective, scalable solutions for treating B‐cell lymphomas.

**Figure 1:**
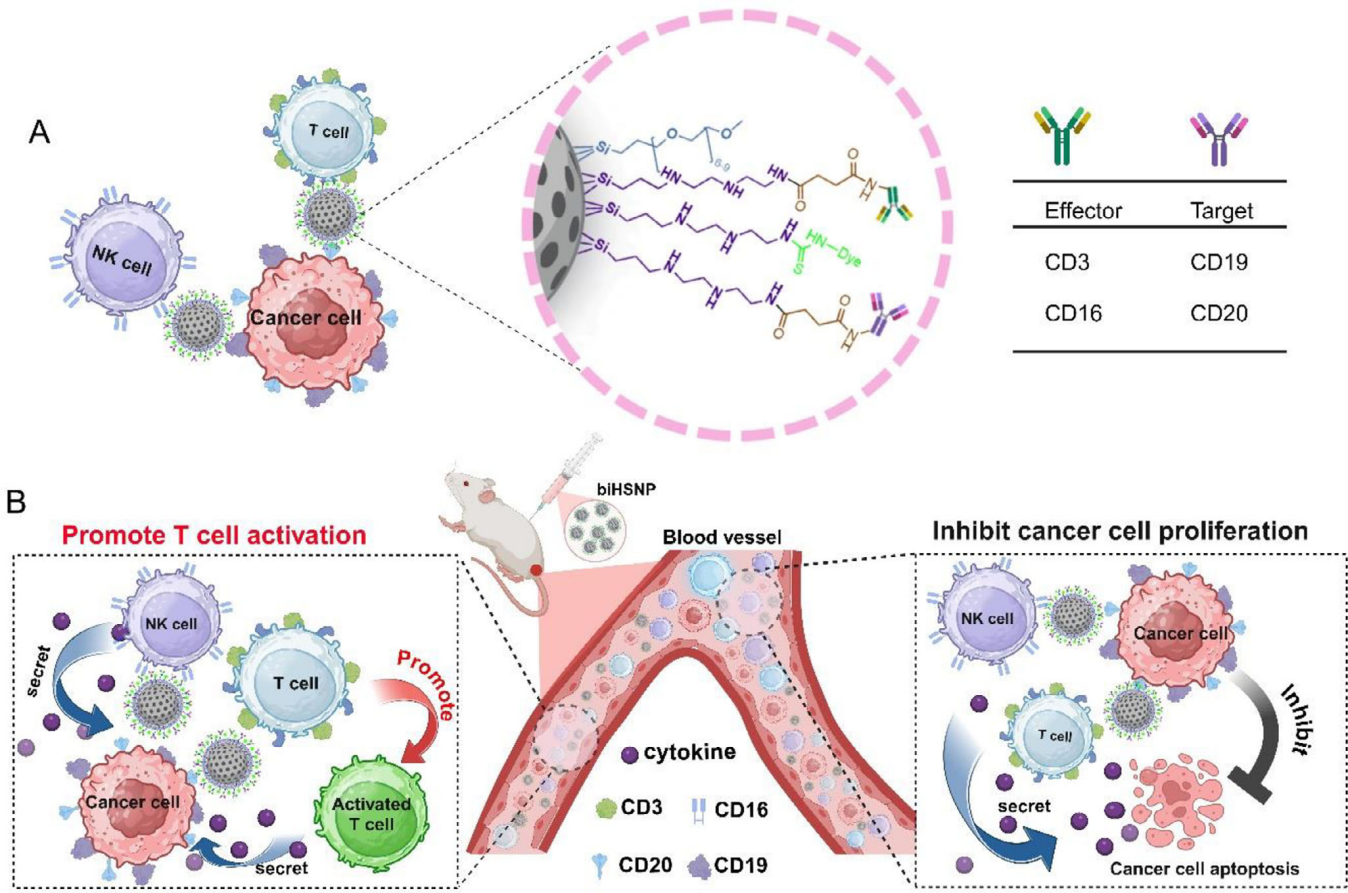
A) This diagram depicts the dual‐targeting strategy of biHSNPs in facilitating tumor cell elimination. Each biHSNP features two functional arms: one specifically binds to a tumor‐associated antigen on lymphoma cells (CD19/CD20), while the other engages and activates immune effector cells (CD3/CD16). By bridging tumor cells and immune cells, this approach enhances immune‐mediated tumor cell lysis, leveraging the immune system's inherent ability to identify and eliminate abnormal cells for precise and efficient cancer cell killing. B) Schematic illustration of the T cell activation and tumor cell death mechanism mediated by biHSNPs.

In this study, we propose a bispecific nanosystem to address the challenges associated with conventional bispecific antibodies. These nanoparticles are biocompatible and biodegradable.^[^ ^16^ ^]^ By functionalizing the nanoparticles with monoclonal antibodies targeting tumor antigens (CD19 and CD20) and immune effector cell receptors (CD3 or CD16), bispecific hollow silica nanoparticles (biHSNPs) mimic the function of bispecific antibodies while reducing immunogenicity. We synthesized a total of four biHSNPs by varying the effector and target antibodies, as well as four monospecific hollow silica nanoparticles (monoHSNPs), in which the surface was functionalized with either a single effector antibody or a single target antibody as controls. This dual‐targeting strategy effectively bridges tumor cells and immune effector cells, facilitating the formation of artificial immunological synapses that enhance immune cell activation and trigger efficient tumor cell lysis (**Figure** **1**). Notably, the synergistic activation of both T cells (CD3^+^) and natural killer (NK) cells (CD16^+^) as immune effectors significantly improved in vitro therapeutic outcomes against B‐cell lymphoma. Furthermore, in vivo studies demonstrated robust anti‐tumor effects and enhanced T cell activation in a mouse xenograft model. This customizable and modular nanoparticle platform represents a promising advancement in cancer immunotherapy. By addressing the limitations of existing strategies, biHSNPs offer a transformative approach to enhance the efficacy, versatility, and precision of cancer treatment.

## 2 Results

### 2.1 Synthesis and Characterization of Antibody‐Functionalized Nanosystem

**Figure 2:**
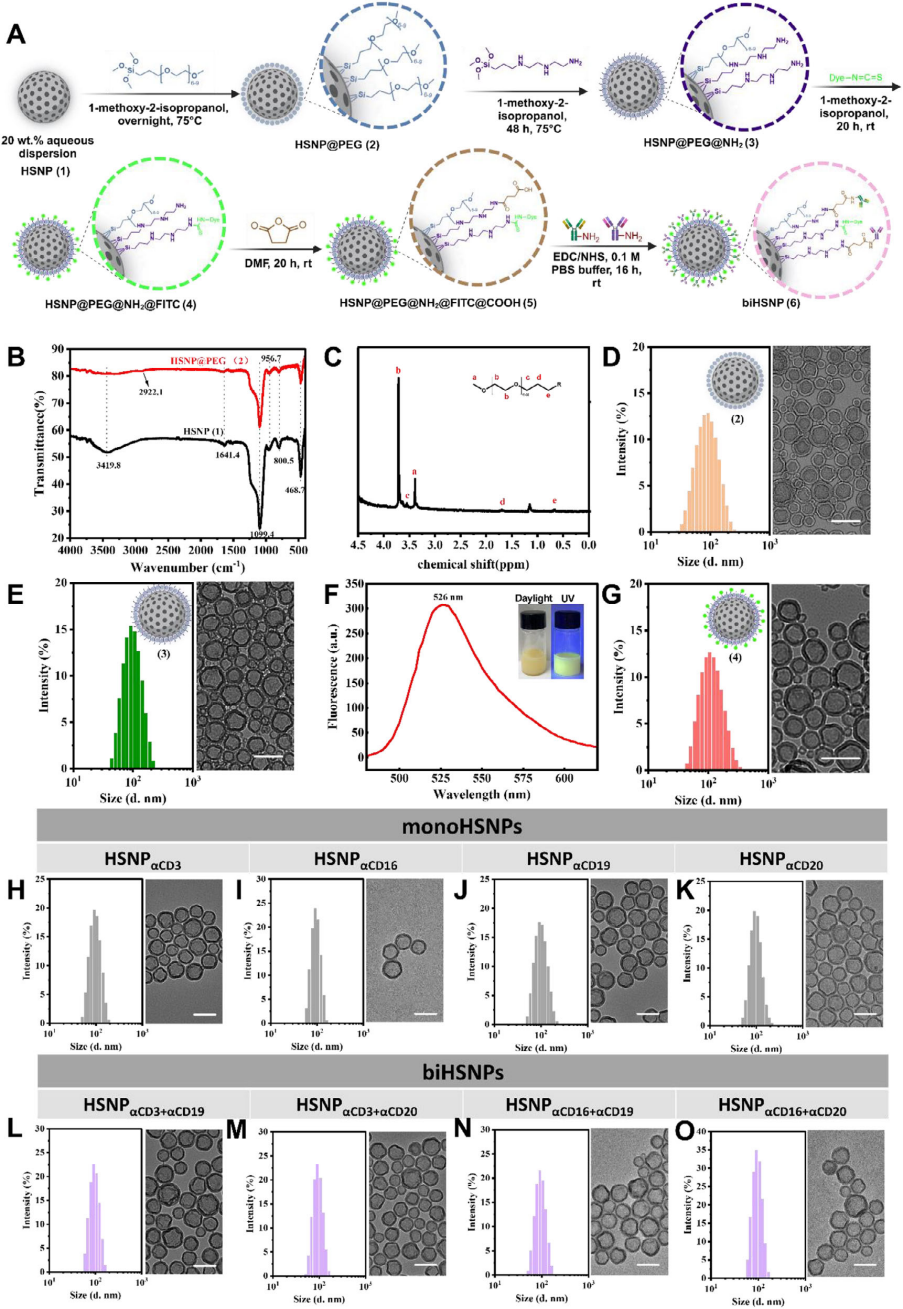
Schematic illustration of the chemical synthesis and characterization of antibody‐conjugated hollow silica nanoparticles. A) Synthetic pathway of PEGylated hollow silica nanoparticles. B) Fourier transform‐infrared spectroscopy (FT‐IR) characterization of HSNP@PEG (2). C) Proton nuclear magnetic resonance (^1^H NMR) characterization of HSNP@PEG. D) DLS and TEM images of HSNP@PEG. E) DLS and TEM images of HSNP@PEG@NH_2_ (3). F) Fluorescence spectrum of HSNP@PEG@NH_2_@FITC (9.62 µm), confirming successful FITC conjugation. G) DLS and TEM images of HSNP@PEG@NH_2_@FITC (4). H–K) DLS and TEM results for monospecific nanoparticles (HSNP_αCD3_, HSNP_αCD16_, HSNP_αCD19_, HSNP_αCD20_). L–O) DLS and TEM results for bispecific nanoparticles (HSNP_αCD3_^+^_αCD19_, HSNP_αCD3_^+^_αCD20_, HSNP_αCD16_^+^_αCD19_, HSNP_αCD16_^+^_αCD20_). All scale bars in the TEM images represent 100 nm. HSNP_αCD3_, HSNP_αCD16_, HSNP_αCD19_ and HSNP_αCD20_: Monospecific HSNPs conjugated with a single type of antibody (anti‐CD3, anti‐CD16, anti‐CD19, or anti‐CD20, respectively). HSNP_αCD3_^+^_αCD19_, HSNP_αCD3_^+^_αCD20_, HSNP_αCD16_^+^_αCD19_ and HSNP_αCD16_^+^_αCD20_: Bispecific HSNPs conjugated with two different types of antibodies (e.g., anti‐CD3 and anti‐CD19). Antibody types are indicated using lowercase Greek letters (e.g., αCD3).

To develop effective biHSNPs for targeting B‐cell lymphomas, we synthesized antibody‐conjugated HSNPs and optimized their properties to ensure immune compatibility and effective targeting (**Figure** **2** and S1, Figures S1–S4, and Tables S1 and S2, Supporting Information). The employed nanoparticles have an average diameter of 75 nm as determined by dynamic light scattering (DLS), with a low polydispersity index (PDI) of 0.159, indicative of uniform size distribution. These characteristics are critical for achieving consistent biodistribution, extended circulation time, and minimized nonspecific aggregation or uptake in vivo (Table S2, Supporting Information). To enhance biocompatibility and prolong the half‐life of the nanoparticles, we modified the HSNP surface with poly(ethylene glycol) (PEG) using methoxy(polyethyleneoxy)propyltrimethoxysilane, as PEGylation is a well‐established strategy to reduce recognition by the mononuclear phagocyte system and immune clearance, allowing the nanoparticles to reach tumor sites more effectively.^[^ ^17^ ^]^ Thermogravimetric analysis (TGA) indicated that the resulting particles contain 16 wt% PEG moieties (Figure 2A–D, Figure S1 and Table S1, Supporting Information). Next, we functionalized the PEGylated HSNPs with amino groups using 3‐[2‐(2‐aminoethylamino)ethylamino]propyl‐trimethoxysilane, enabling the stable conjugation of antibodies and fluorescent markers (Figure 2A,E). A ninhydrin assay confirmed an amino substitution degree of 15.75 µmol g^−1^, indicating sufficient functional groups for subsequent antibody attachment (Figure S2, Supporting Information). To verify the successful modification, fluorescein isothiocyanate (FITC) was conjugated to the nanoparticle surface, achieving a substitution degree of 0.125 µmol g^−1^ (Figure 2F–G, Figures S3 and S14, Supporting Information). For both FITC‐conjugated and non‐conjugated nanoparticles, the amino groups were reacted with succinic anhydride to generate carboxylic acid groups, which were activated using 1‐ethyl‐3‐(3‐dimethylaminopropyl) carbodiimide and N‐hydroxysuccinimide (Figure S4, Supporting Information). Monoclonal antibodies were subsequently conjugated to the activated carboxylate groups via amide bond formation (Figure 2H–O and Table S2, Supporting Information). The surface antibody densities were determined to be 4.08 µg mg^−1^ for HSNP_αCD3_^+^_αCD20_, while a concentration of 4.81 µg mg^−1^ was determined for HSNP_αCD3_^+^_αCD19_ (Figure S5, Supporting Information).

We synthesized four types of biHSNP constructs for immunotherapy of B‐cell lymphomas, incorporating two different monoclonal antibodies on the nanoparticle surface. For control experiments, we also generated prepared four monoHSNPs, and each one was conjugated with a single antibody (Figure 2H–K). The selected monoclonal antibodies included Anti‐CD19 antibody (αCD19) and Anti‐CD20 antibody (αCD20), which bind tumor‐associated antigens, and anti‐CD3 antibody (αCD3) and anti‐CD16 antibody (αCD16), which target immune effector cell receptors. The biHSNPs HSNP_αCD3_^+^_αCD19_ and HSNP_αCD3_^+^_αCD20_ were designed to redirect T‐cells to malignant B‐cells by simultaneously binding CD3 on T‐cells and CD19 or CD20 on tumor cells. This dual‐targeting mechanism is expected to trigger T‐cell activation and cytokine secretion, leading to tumor cell lysis^[^ ^13^, ^15^, ^18^ ^]^ (Figure 2L–M). Similarly, HSNP_αCD16_^+^_αCD19_ and HSNP_αCD16_^+^_αCD20_ were designed to activate NK cells and other CD16‐expressing immune cells. These biHSNPs may bind both CD16 on immune cells and CD19/CD20 on tumor cells, enabling specific immune cell‐mediated cytotoxicity against B‐cell lymphomas (Figure 2N–O).

### 2.2 Bispecific Nanosystem Functionality in Immune‐Tumor Cell Interaction

**Figure 3:**
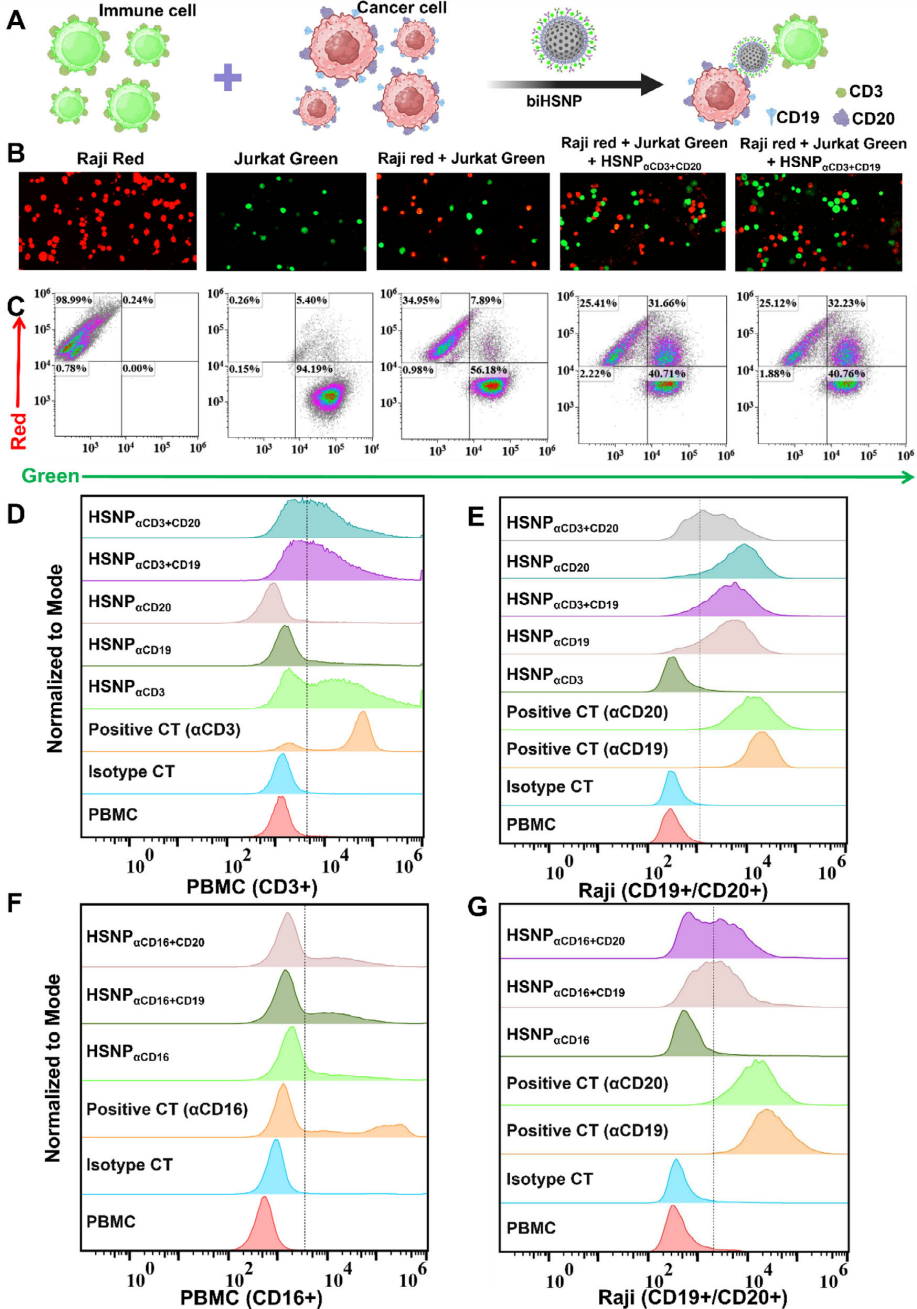
Binding specificity and cell–cell linkage induced by biHSNPs. Target cells were incubated with FITC‐labeled, antibody‐coated HSNPs at 4 °C for 30 min, followed by flow cytometry analysis to confirm binding specificity. A–C) Depict cell–cell linkage induced by biHSNPs: A) Schematic illustration of cell‐cell complex formation mediated by biHSNPs. B) Confocal microscopy images showing stable junctional complexes between immune effector cells and tumor cells facilitated by HSNP_αCD3_^+^_αCD19_ and HSNP_αCD3_^+^_αCD20_. C) Flow cytometry analysis of cell–cell complexes between pre‐stained Jurkat (green, CellTracker Green CMFDA) and Raji cells (red, CellTracker Red CMTPX), demonstrating effective linkage by biHSNPs. D) Verification of biHSNPs binding to CD3^+^ cells using PBMCs as the CD3^+^ cell line. E) Verification of biHSNPs binding to CD19^+^/CD20^+^ cells using Raji cells as the target. F) Verification of biHSNPs binding to CD16^+^ cells using PBMCs as the CD16^+^ cell line. G) Verification of biHSNPs binding to CD19^+^/CD20^+^ cells using Raji cells as the target.

To assess the functionality of biHSNPs in mediating immune‐tumor cell interactions, we evaluated their ability to form stable complexes between immune effector cells and tumor cells (**Figure** **3**A–C). Jurkat T cells (CD3^+^) were prelabeled with a green fluorescent dye, while Raji B cells (CD19^+^/CD20^+^), a well‐characterized human B‐cell lymphoma cell line, were labeled with a red fluorescent dye. Upon addition of HSNP_αCD3_^+^_αCD19_ or HSNP_αCD3_^+^_αCD20_, confocal microscopy revealed significantly enhanced cellular bridging, indicating efficient facilitation of immune‐tumor cell engagement (Figure 3B). Quantification by flow cytometry confirmed the formation of cell‐cell complexes. HSNP_αCD3_^+^_αCD19_ and HSNP_αCD3_^+^_αCD20_ exhibited cell‐cell linkage rates of 32.23% and 31.66%, respectively, compared to only 7.89% in control samples without biHSNPs (Figure 3C). These results highlight the ability of biHSNPs to effectively bridge immune effector cells and tumor cells, forming stable complexes that are critical for targeted immune cell activation and tumor lysis.

### 2.3 Specificity and Binding Efficiency of Bispecific Nanosystems

The specificity of antibody‐conjugated HSNPs was evaluated using peripheral blood mononuclear cells (PBMCs) as immune effector cell sources (CD3^+^ and CD16^+^) and Raji cells as tumor cell targets (CD19^+^/CD20^+^). FITC‐labeled antibody‐conjugated HSNPs were incubated with their respective antigen‐positive cell lines, and binding specificity was analyzed by flow cytometry and confocal microscopy (Figure 3D–G and Figures S6–S9, Supporting Information). For HSNP_αCD3_^+^_αCD19_ and HSNP_αCD3_^+^_αCD20_, flow cytometry demonstrated a significant rightward fluorescence shift in PBMCs treated with these biHSNPs, confirming successful αCD3 antibody conjugation. In contrast, HSNP_αCD19_ and HSNP_αCD20_ alone showed no significant fluorescence increase in PBMCs (Figure 3D and Figure S7A, Supporting Information). Moreover, HSNP_αCD3_, HSNP_αCD3_^+^_αCD19_ and HSNP_αCD3_^+^_αCD20_ incubated with Jurkat cells as the CD3^+^ cell line showed a rightward fluorescence shift as well (Figure S6, Supporting Information). Similarly, FITC‐labeled HSNP_αCD19_, HSNP_αCD20_, HSNP_αCD3_^+^_αCD19_, and HSNP_αCD3_^+^_αCD20_ exhibited significantly increased fluorescence with Raji cells as compared to the control, verifying the successful conjugation of αCD19 and αCD20 antibodies to the nanoparticle surface (Figure 3E and Figure S7B, Supporting Information). Moreover, confocal microscopy images of HSNP_αCD3_^+^_αCD19_ and HSNP_αCD3_^+^_αCD20_ incubated with either Jurkat as CD3^+^ or Raji as CD19^+^/CD20^+^ positive cell lines showed significant green fluorescence, implying the successful binding of the biHSNPs of the corresponding cell lines (Figure S8, Supporting Information).

The binding specificity of HSNP_αCD16_^+^_αCD19_ and HSNP_αCD16_^+^_αCD20_ was further validated using PBMCs as CD16^+^ immune effector cells and Raji cells as tumor cell targets. In PBMCs, a rightward fluorescence shift confirmed the successful conjugation of αCD16 to HSNP_αCD16_, HSNP_αCD16_^+^_αCD19_, and HSNP_αCD16_^+^_αCD20_ (Figure 3F and Figure S7C, Supporting Information). Raji cells treated with HSNP_αCD16_^+^_αCD19_ and HSNP_αCD16_^+^_αCD20_ displayed strong fluorescence signals, further validating the conjugation of αCD19 and αCD20 antibodies to the nanoparticle surface (Figure 3G and Figure S7D, Supporting Information). Confocal microscopy further demonstrated efficient binding of HSNP_αCD16_^+^_αCD19_ and HSNP_αCD16_^+^_αCD20_ to Raji cells, as indicated by the bright green fluorescence observed in treated cells (Figure S9, Supporting Information).

Collectively, these results demonstrate that HSNP_αCD3_^+^_αCD19_, HSNP_αCD3_^+^_αCD20_, HSNP_αCD16_^+^_αCD19_, and HSNP_αCD16_^+^_αCD20_ bind specifically to their target antigens on PBMCs and Raji cells. The fluorescence intensity achieved with these biHSNPs is comparable to that observed with high‐affinity monoclonal antibodies, confirming the effectiveness of the conjugation strategy. These findings support the use of biHSNPs as a robust and modular platform for targeted immunotherapy, capable of bridging immune effector cells and tumor cells for enhanced therapeutic outcomes.

### 2.4 In Vitro Cytotoxicity Assays and Cytokine Release Measurements

**Figure 4:**
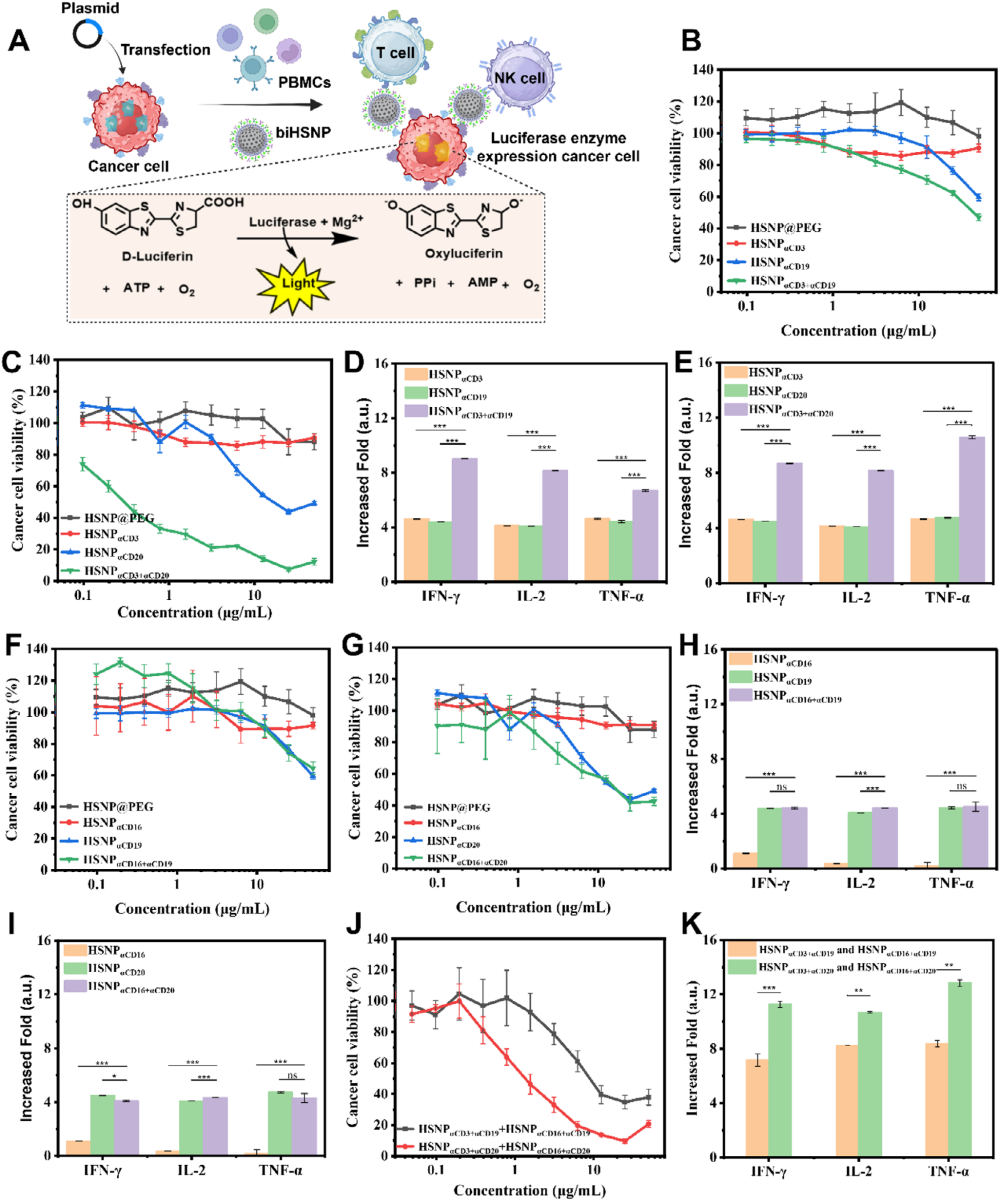
A) Cytotoxicity and cytokine release assays of biHSNPs in luciferase‐transfected Raji cells. B) In vitro cytotoxicity of HSNP@PEG, HSNP_αCD3_, HSNP_αCD19_, and bispecific HSNP_αCD3_^+^_αCD19_. C) In vitro cytotoxicity of HSNP@PEG, HSNP_αCD3_, HSNP_αCD20_, and bispecific HSNP_αCD3_^+^_αCD20_. D,E) Cytokine secretion levels (IFN‐γ, IL‐2, and TNF‐α) for HSNP_αCD3_, HSNP_αCD19_, and HSNP_αCD3_^+^_αCD19_; and HSNP_αCD20_ and HSNP_αCD3_^+^_αCD20_. F) In vitro cytotoxicity of HSNP@PEG, HSNP_αCD16_, HSNP_αCD19_, and bispecific HSNP_αCD16_^+^_αCD19_. G) In vitro cytotoxicity of HSNP@PEG, HSNP_αCD16_, HSNP_αCD20_, and bispecific HSNP_αCD16_^+^_αCD20_. H,I) Cytokine secretion levels (IFN‐γ, IL‐2, and TNF‐α) for HSNP_αCD16_, HSNP_αCD19_, HSNP_αCD16_^+^_αCD19_, HSNP_αCD20_, and HSNP_αCD16_^+^_αCD20_. J) Cytotoxicity of combined bispecific nanoparticle treatments: HSNP_αCD3_^+^_αCD19_ with HSNP_αCD16_^+^_αCD19_, and HSNP_αCD3_^+^_αCD20_ with HSNP_αCD16_^+^_αCD20_. K) In vitro cytotoxicity of HSNP_αCD3_^+^_αCD19_ combined with HSNP_αCD16_^+^_αCD19_, and HSNP_αCD3_^+^_αCD20_ combined with HSNP_αCD16_^+^_αCD20_. Data are presented as mean ± SD (*n* ≥ 3), Statistical differences in B–I) were analyzed by One‐way ANOVA and K) were analyzed by Student's t‐test and the statistical significance is indicated as **P ≤ 0.01 and ***P ≤ 0.001.

To investigate whether biHSNPs could mediate targeted cytotoxicity, we evaluated their ability to redirect mixed immune effector cells (PBMCs) toward CD19^+^/CD20^+^ target cells. A luminescence assay quantified the cytotoxic effects, using luciferase‐transfected Raji cells as tumor targets and IL‐2‐preactivated PBMCs as enriched CD3^+^/CD16^+^ immune effector cells sources to enable targeted immune activation (**Figure** **4**). Various nanoparticle formulations, including HSNPs, PEGylated HSNPs (HSNP@PEG), monoHSNPs and biHSNPs, were co‐cultured with Raji cells at a 20:1 effector‐to‐target ratio for varying incubation times. Luminescence data confirmed a dose‐dependent decrease in cell viability with unmodified HSNPs, particularly at concentrations exceeding 25 µg mL^−1^, where substantial cytotoxicity was observed (Figure S10A, Supporting Information). PEGylation significantly reduced this cytotoxicity, with HSNP@PEG maintaining minimal impact on cell viability, thereby establishing a safe working concentration range of 0–50 µg mL^−1^ for subsequent experiments (Figure S10B, Supporting Information).

#### 2.4.1 Evaluation of CD3‐Targeting biHSNPs

Dual antibody‐conjugated biHSNPs, such as HSNP_αCD3_^+^_αCD19_ and HSNP_αCD3_^+^_αCD20_ (Figure 4B,C, green lines), exhibited markedly higher cytotoxicity compared to monospecific nanoparticles, including HSNP_αCD3_, HSNP_αCD19_, and HSNP_αCD20_ (Figure 4B,C, red and blue lines). Interestingly, HSNP_αCD20_ demonstrated slightly greater cytotoxicity than HSNP_αCD19_ under similar conditions, highlighting the enhanced tumor‐killing efficiency of αCD20 targeting alone. Cytotoxicity assays with pure antibodies further validated this observation, as αCD20 consistently outperformed αCD19 in killing Raji cells in vitro (Figure S11, Supporting Information). Specificity testing on CD19/CD20‐negative L‐428 cell lines transfected with a luciferase reporter gene (L‐428_luc cells) revealed minimal cytotoxicity with all nanoparticle formulations, confirming the tumor antigen‐specific nature of biHSNP‐mediated cytotoxicity (Figure S12, Supporting Information). To quantify immune activation, we measured cytokine secretion IFN‐γ (Interferon gamma), IL‐2 (Interleukin 2), and TNF‐α (tumor necrosis factor alpha) in co‐cultures using enzyme‐linked immunosorbent assay (ELISA). Monospecific HSNP_αCD3_, HSNP_αCD19_, and HSNP_αCD20_ showed a fourfold increase in cytokine release compared to controls, while bispecific HSNP_αCD3_^+^_αCD19_ and HSNP_αCD3_^+^_αCD20_ induced an eight‐fold increase (Figure 4D–E). These results indicate that the dual targeting mechanism significantly enhances immune effector activation.

#### 2.4.2 Evaluation of CD16‐Targeting biHSNPs

We further assessed the performance of CD16‐targeting biHSNPs (HSNP_αCD16_^+^_αCD19_ and HSNP_αCD16_^+^_αCD20_) designed to redirect CD16^+^ immune cells, such as NK cells and monocytes, toward tumor cells. As shown in Figure 4F,G, these nanoparticles exhibited cytotoxicity comparable to monospecific HSNP_αCD19_ and HSNP_αCD20_, but their efficacy was lower than that of CD3‐targeting biHSNPs. PEGylated and FITC‐labeled HSNPs displayed negligible toxicity against Raji cells, reaffirming that the observed cytotoxicity was antibody‐mediated rather than due to non‐specific effects. Specificity assays using KMH2 cell lines transfected with a luciferase reporter gene (KMH2_luc cells) (CD19/CD20‐negative) confirmed minimal off‐target effects for CD16‐targeting biHSNPs (Figure S13, Supporting Information). ELISA measurements showed a 1.5‐ to 2‐fold increase in cytokine secretion (IFN‐γ, IL‐2, and TNF‐α) with CD16‐targeting biHSNPs compared to controls, though this response was less pronounced than with CD3‐targeting biHSNPs (Figure 4H–I).

#### 2.4.3 Synergistic Effects of Combined biHSNP Treatments

The combination of CD3‐ and CD16‐targeting biHSNPs (e.g., HSNP_αCD3_^+^_αCD19_ with HSNP_αCD16_^+^_αCD19_) demonstrated a synergistic increase in cytotoxicity and cytokine secretion compared to individual treatments. For example, combinations of HSNP_αCD3_^+^_αCD19_ with HSNP_αCD16_^+^_αCD19_ and HSNP_αCD3_^+^_αCD20_ with HSNP_αCD16_^+^_αCD20_ resulted in significantly higher tumor cell lysis and cytokine levels, with approximately 7‐ and 12‐fold increases in IFN‐γ, IL‐2, and TNF‐α secretion, respectively (Figure 4J–K). The synergistic cytotoxicity and increased cytokine production from combined CD3‐ and CD16‐targeting biHSNPs arise from the simultaneous activation of T cells and NK cells. CD3 activation triggers T cell responses,^[^ ^19^ ^]^ while CD16 activation boosts NK cell activity, promoting antibody‐dependent cellular cytotoxicity (ADCC) and cytokine release.^[^ ^20^ ^]^ This dual activation enhances immune responses through IL‐2‐mediated NK cell support and NK‐derived cytokines that amplify T cell activity. Co‐delivery of both biHSNPs further strengthens immune‐tumor interactions, amplifying cytotoxicity and cytokine production.

Overall, biHSNPs demonstrated potent, dose‐dependent cytotoxicity against CD19^+^/CD20^+^ cells, with HSNP_αCD3_^+^_αCD19_ and HSNP_αCD3_^+^_αCD20_ exhibiting superior efficacy compared to their CD16‐targeting counterparts. The enhanced immune activation and cytokine release observed with dual‐targeting biHSNPs underscore their therapeutic potential. These results position HSNP_αCD3_^+^_αCD19_ and HSNP_αCD3_^+^_αCD20_ as strong candidates for further in vivo evaluation, offering a promising pathway for advancing targeted immunotherapy against B‐cell lymphomas.

### 2.5 In Vivo Tumor Suppression by biHSNPs

**Figure 5:**
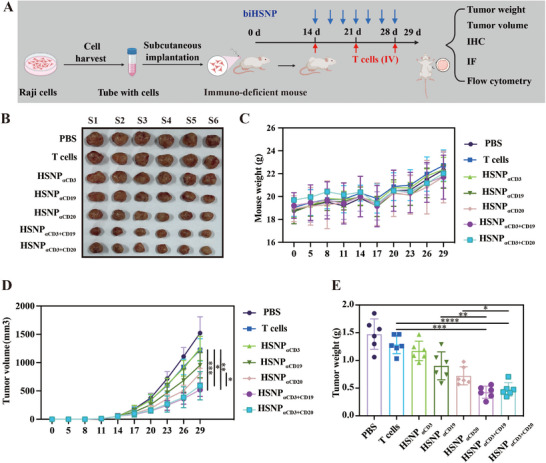
Inhibitory effects of biHSNPs on tumor growth in vivo. A) Schematic representation illustrating the anticancer mechanism of biHSNPs in a xenograft mouse model. B) Representative images of mice bearing tumors developed from Raji cell xenografts. C) Body weights of mice across experimental groups, demonstrating the tolerability of biHSNPs treatments. D) Tumor volumes and E) tumor weights were measured at 3 d intervals, highlighting the significant tumor‐suppressive effects of biHSNPs. Statistical differences are analyzed by using One‐way ANOVA. Statistical significance is indicated as *P ≤ 0.05, **P ≤ 0.01, ***P ≤ 0.001 and ****P ≤ 0.0001.

The antitumor efficacy of biHSNPs was evaluated in vivo using a nude mouse xenograft model, designed to mimic human B‐cell lymphoma. Tumor‐bearing models were established by subcutaneously injecting Raji cells into nude mice, followed by regular monitoring of tumor volume and body weight every 3 d for 29 d (**Figure** **5**A,B).

To determine the optimal therapeutic dose, we administered HSNP_αCD3_^+^_αCD19_ at 1, 2, and 4 mg kg^−1^. Tumor volumes were significantly reduced in the 2 and 4 mg kg^−1^ groups, with both doses inducing comparable antitumor effects. Importantly, CD19 expression levels in tumors were elevated in these treatment groups, suggesting effective tumor targeting by biHSNPs (Figure S15A—C, Supporting Information). Based on these findings, the 2 mg kg^−1^ dose was selected for subsequent experiments due to its balance of efficacy and tolerability. No significant changes in body weight were observed across the experimental groups, indicating that biHSNP treatment was well tolerated (Figure 5C). BiHSNP treatment, particularly with HSNP_αCD3_^+^_αCD19_ and HSNP_αCD3_^+^_αCD20_, significantly suppressed tumor growth, as reflected in both tumor size and weight reductions compared to controls, including groups treated with T cells alone (Figure 5D,E). Notably, biHSNPs targeting both CD3 and CD19/CD20 demonstrated superior efficacy over monospecific nanoparticles (HSNP_αCD19_ and HSNP_αCD20_), underscoring the advantages of dual‐targeting strategies in vivo. These results establish the potent antineoplastic properties of biHSNPs, which effectively inhibit tumor progression while maintaining a favorable safety profile. The ability of biHSNPs to simultaneously engage immune effector cells and tumor‐specific antigens offers a promising and innovative approach to targeted cancer immunotherapy, particularly for B‐cell lymphomas.

### 2.6 Activation of Tumor‐Infiltrating T Cells and Modulation of the Tumor Microenvironment

**Figure 6:**
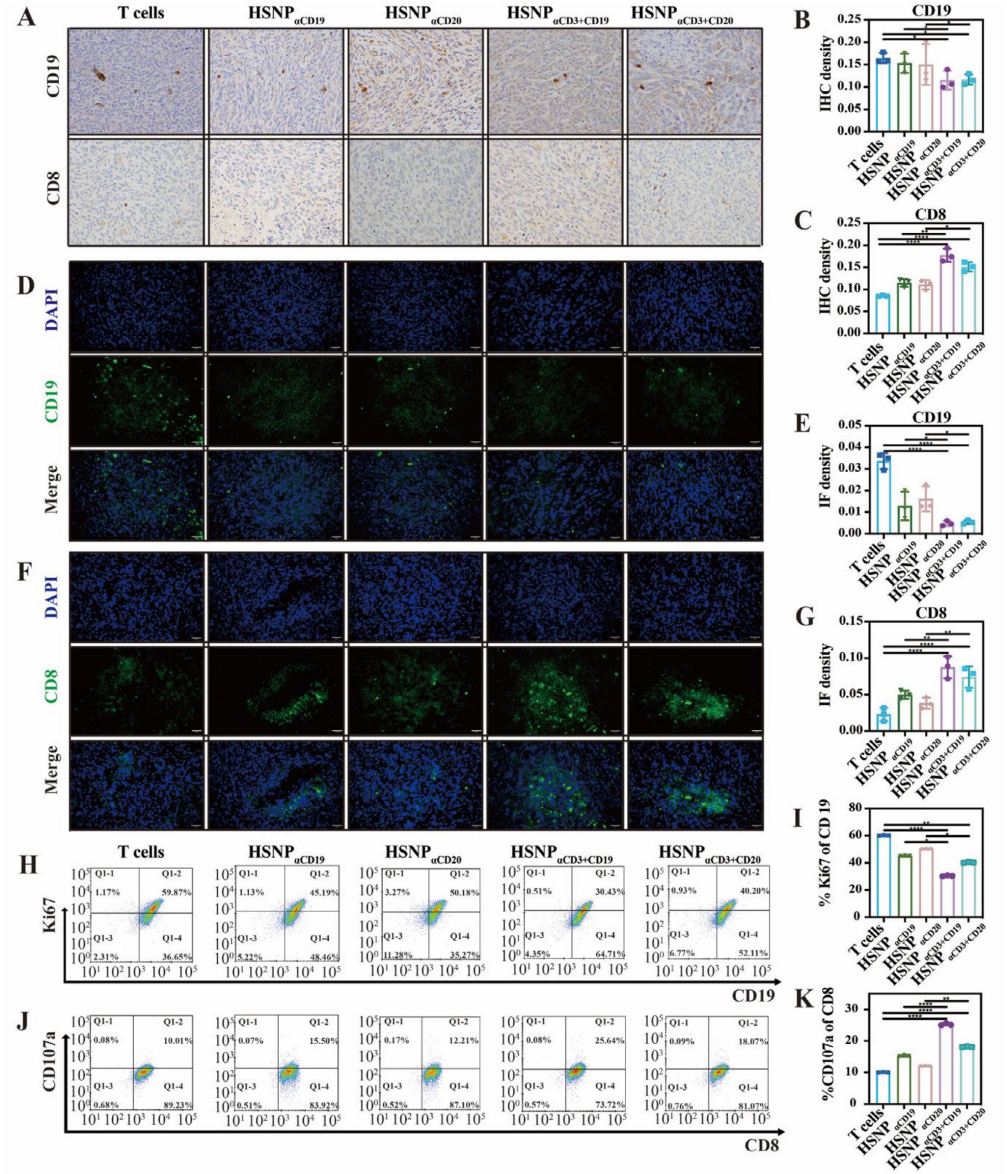
Enhanced T‐cell activation mediated by biHSNPs. A) Representative Quantitative analysis of CD19^+^ and CD8^+^ cell populations based on IHC. D,F) Representative immunofluorescence (IF) images showing the distribution of CD19^+^ Raji cells and CD8^+^ T cells. E–G) Quantitative analysis of CD19^+^ and CD8^+^ cells based on IF staining. H–J) Representative flow cytometry plots illustrating; I) Ki67^+^ expression in Raji cells and K) CD107a expression in CD8^+^ T cells across treatment groups. Statistical differences are analyzed by using One‐way ANOV and statistical significance is indicated as *P ≤ 0.05, **P ≤ 0.01 and ****P ≤ 0.0001.

To elucidate the mechanisms underlying tumor suppression mediated by biHSNPs, we performed immunohistochemistry (IHC) and immunofluorescence (IF) analyses to assess the impact of biHSNP treatment on tumor‐infiltrating immune cells and the tumor microenvironment. A significant reduction in the CD19 level was observed in tumors from the HSNP_αCD3_^+^_αCD19_ and HSNP_αCD3_^+^_αCD20_ treatment groups, reflecting effective targeting and suppression of Raji cells (**Figure** **6**A,B). Concurrently, a lower CD8 expression level was detected in these treatment groups compared to the T cell control group, suggesting that biHSNPs modulate immune cell populations within the tumor microenvironment (Figure 6A,C). These findings were corroborated by IF analysis, which confirmed the suppression of CD19^+^ tumor cells and altered CD8^+^ T cell distribution (Figure 6D–G). To further investigate T cell activation in vivo, we employed flow cytometry to analyze markers indicative of T cell proliferation and activation. Tumor samples from HSNP_αCD3_^+^_αCD19_ and HSNP_αCD3_^+^_αCD20_‐treated mice showed a notable decrease in Ki67 expression, a marker of tumor cell proliferation, indicating reduced tumor growth (Figure 6H,I). Furthermore, we evaluated CD107a, a marker of T cell degranulation and activation, in intratumoral CD8^+^ T cells. Our results demonstrated a significant increase in CD8^+^ CD107a^+^ T cells in the HSNP_αCD3_^+^_αCD19_ and HSNP_αCD3_^+^_αCD20_ groups compared to those treated with monospecific HSNP_αCD19_ or HSNP_αCD20_, highlighting the enhanced activation of tumor‐infiltrating T cells induced by biHSNP treatment (Figure 6J,K). These results underscore the dual functionality of biHSNPs: not only do they effectively suppress tumor cell proliferation, but they also reshape the tumor microenvironment by activating and expanding tumor‐infiltrating T cells. This modulation amplifies the antitumor immune response, demonstrating the potential of biHSNPs as a robust strategy for enhancing cancer immunotherapy through targeted immune activation.

## 3 Discussion

This study introduces a bispecific antibody‐functionalized nanosystem as a transformative platform for precision immunotherapy in aggressive B‐cell lymphomas. By addressing critical limitations of conventional immunotherapies—such as short antibody half‐life, high treatment costs, and immune escape mechanisms—biHSNPs represent a significant advancement in nanomedicine. Through simultaneous targeting of tumor‐associated antigens (CD19/CD20) and immune effector cell receptors (CD3/CD16), biHSNPs act as a bridge between immune and tumor cells, facilitating the formation of artificial immunological synapses. This dual‐targeting strategy effectively triggers robust tumor‐specific cytotoxicity and cytokine secretion, as demonstrated in both in vitro and in vivo models, positioning biHSNPs as a promising tool for developing precision therapies for hematologic malignancies.

The synthesis and characterization of biHSNPs highlighted the critical role of PEGylation in enhancing their biocompatibility, reducing immune clearance, and extending circulation time. These properties, coupled with controlled antibody conjugation, ensured efficient delivery to tumor sites and robust targeting efficacy. Moreover, the uniform size distribution of the nanoparticles minimized nonspecific uptake, improving reproducibility and addressing persistent challenges in nanoparticle‐based therapeutics. The functionality of biHSNPs in bridging immune effector and tumor cells was validated using confocal microscopy and flow cytometry, which confirmed the formation of stable immune‐tumor cell complexes. The dual‐targeting strategy—achieved through conjugating tumor antigens (CD19 or CD20) with immune cell‐activating receptors (CD3 or CD16)—enhanced immune engagement, efficiently triggering tumor cell lysis. This mechanism underscores the potential of biHSNPs to amplify immune responses by facilitating precise recruitment of immune cells to malignant targets. The potential mechanism of the CD3‐targeting biHSNPs is to activate T cells, and mediate potent cytotoxicity through granzyme^[^ ^21^ ^]^ and perforin release and pro‐inflammatory cytokine production^[^ ^21^ ^]^ and undergo clonal expansion.^[^ ^22^ ^]^ However, CD16‐targeting biHSNPs mediate ADCC and may lack proliferative capacity,^[^ ^23^ ^]^ leading to the weak cytotoxicity than CD3‐targeting biHSNPs. Notably, we employed a nude mouse xenograft model to clearly elucidate the T cell‐mediated cytotoxic effects induced by biHSNPs against tumor cells. Interestingly, we found the CD8^+^T cells were proliferated (Figure 6F,G) and activated by biHSNPs, providing the considerable promise for clinical application. One of the most compelling aspects of this study is the versatility of the bispecific nanosystem platform. In vitro cytotoxicity assays demonstrated that biHSNPs induced significant tumor‐specific cytotoxicity and cytokine release, including IFN‐γ and TNF‐α, surpassing the efficacy of monospecific nanoparticles. Activated NK cells may secrete IFN‐γ and TNF‐α, which enhance antigen presentation and facilitate T cell activation, while activated T cells produce IL‐2 and other cytokines that sustain NK cell cytotoxic function. Different with the application of delivering CD4/CD6 inhibitors as molecularly targeted immunotherapy, the dual‐targeting approach not only amplified immune responses but also showcased modular adaptability, allowing rapid customization to target diverse cancer‐associated antigens or immune cell types. This flexibility highlights the potential of biHSNPs to be tailored for a wide range of applications across both hematological and solid malignancies.

Future studies should focus on several key areas to facilitate the clinical translation of bispecific nanosystems. Comprehensive preclinical evaluations are necessary to assess long‐term safety, biodistribution, and pharmacokinetics. Further optimization of nanoparticle design and antibody loading capacity could enhance therapeutic efficacy while minimizing off‐target effects. Additionally, combining biHSNPs with complementary therapeutic modalities—such as chemotherapy, immune checkpoint inhibitors, amino acid metabolism of immune cells,^[^ ^24^ ^]^ or adoptive cell therapies—may yield synergistic effects and broaden their therapeutic scope.

## 4 Conclusion

In conclusion, this study establishes a bispecific nanosystem as a novel platform for targeted cancer immunotherapy in aggressive B‐cell lymphomas. By simultaneously engaging tumor‐associated antigens (CD19/CD20) and immune effector cell receptors (CD3/CD16), our bispecific nanosystem effectively bridges immune and tumor cells, inducing robust tumor‐specific cytotoxicity and cytokine secretion. The enhanced biocompatibility and prolonged circulation time achieved through PEGylation, combined with precise antibody conjugation, enabled efficient and specific tumor targeting. Both in vitro and in vivo results demonstrated the efficacy of biHSNPs in inducing immune‐mediated tumor cell lysis, suppressing tumor growth, and overcoming tumor immune evasion, addressing key limitations of conventional immunotherapies. Furthermore, the modular and adaptable design of the bispecific nanosystem allows for rapid customization to target a wide range of cancer types, broadening its therapeutic applications. Future directions include optimizing nanoparticle design, evaluating long‐term safety profiles, and integrating the bispecific nanosystem with complementary therapeutic strategies to enhance its clinical efficacy. Overall, the bispecific nanosystem represents a significant advancement in nanomedicine, offering a robust, precise, and flexible approach to harnessing the immune system for future translational research and clinical development.

## 5 Experimental Section

### Ethical Regulations

The research presented here complies with all relevant ethical regulations. All experiments involving animals were reviewed and approved (IIT‐2022‐28) by the institutional ethics committee of Guangxi Academy of Medical Sciences, China.

### Materials and Reagents

3‐[2‐(2‐Aminoethylamino)ethylamino]propyl‐trimethoxysilane (95%), fluorescein isothiocyanate (97%), succinic anhydride (98%), N,N‐dimethylformamide (99.5%), N‐hydroxysuccinimide (99%), 1‐(3‐dimethylaminopropyl)‐3‐ethylcarbodiimide hydrochloride (99%), and sodium hydroxide (97%) were purchased from Meryer (Shanghai, China). 2‐(Morpholin‐4‐yl)ethane‐1‐sulfonic acid (98%), potassium chloride (99%), phosphoric acid, monosodium salt (97%), phosphoric acid, dipotassium salt (98%), triketohydindene hydrate (97%), disodium ethylenediamine tetraacetate (99%), potassium acetate (99%), deuterium‐oxide (≥99%), and phenolphthalein (99%) were provided by Tianjin Heowns Biochem Co., Ltd. (Tianjin, China). Silica hollow nanospheres in water (20%) were purchased from Dilato Materials Technology Co., Ltd. (China, Ningbo). Methoxy(polyethyleneoxy)propyltrimethoxysilane (80%) was purchased from Gelest, Inc. (Pennsylvania, USA). 1‐Methoxy‐2‐propanol (≥99.5%) was acquired from Titan Scientific (China, Shanghai). Sodium chloride (99.9%) was purchased from Shanghai Bide Technology Co., Ltd. (Shanghai, China). Sodium bicarbonate (≥99.8%) was obtained from Shanghai Aladdin Chemistry Co., Ltd. (Shanghai, China). Potassium bromide was provided by Tianjin Guangfu Fine Chemical Research Institute (China, Tianjin). Acetic acid (≥99.7%) was provided by Tianjin Jiangtian Chemical Technology Co., Ltd. (Tianjin, China).

Bovine serum albumin (BSA) was provided by Beyotime (Shanghai, China). RPMI‐1640 medium, Penicillin streptomycin solution and phosphate buffered saline were acquired from Melone Pharmaceutical Co., Ltd. (China, Dalian). Human peripheral blood mononuclear cells (PBMCs) were provided by HOPE BIOLOGY Co., Ltd (Chengdu, China), and the T cells were isolated from the PBMC using the Pan T Cell Isolation Kit (92‐01‐0262, Xinbio) according to the manufacturer's instructions. Fetal bovine serum (FBS) was obtained from Genetimes Technology, Inc. (Shanghai, China). Anti‐CD16 antibody, Tafasitamab (alternative name: anti‐CD19 antibody, catalog no.: DHD10803, species reactivity: human) and Muromonab (alternative name: OKT3 antibody, catalog No.: DHC27701, species reactivity: human) were acquired from AntibodySystem (Strasbourg, France). Rituximab injection (alternative name: anti‐CD20 antibody, lot number: BD20231114, species reactivity: Human) was purchased from Jiangsu Chia Tai‐Tianqing Pharmaceutical Co., Ltd. (Jiangsu, China). FITC anti‐human CD19 antibody[CB19] (catalog No.: E‐AB‐F1004C, species reactivity: human), FITC anti‐human CD20 antibody[BCA/B20] (Catalog No.: E‐AB‐F1045C, species reactivity: human) and FITC anti‐human CD3 antibody[OKT‐3] (catalog No.: E‐AB‐F1001C, species reactivity: human) were obtained from Elabscience Biotechnology Co.,Ltd. (China, Wuhan).

The MES buffer solution of pH 6.8 was prepared by dissolving 0.1 m 2‐morpholinoethanesulfonic acid monohydrate in deionized water freshly before each synthesis and was verified using an inoLab pH Level 1 pH meter.

### Synthesis of HSNP@PEG (2) and HSNP@PEG@NH_2_ (3)

20.00 g of silica hollow nanocapsules (20% dispersion in water) were added to a 100 mL two‐neck round‐bottom flask, followed by the addition of 6 mL of 1‐methoxy‐2‐propanol. Subsequently, 3.5 g of 3‐[methoxy(polyethyleneoxy)6‐9]propyltrimethoxysilane and 28 mL of 1‐methoxy‐2‐propanol were introduced to the reaction mixture. The solution was stirred at 70 °C overnight. A portion of the reaction product HSNP@PEG (2) was collected for characterization experiments. The remaining reaction mixture was purged with nitrogen gas for 30 minutes. Next, 0.19 g of N‐(6‐aminohexyl)aminopropyltrimethoxysilane (M = 278) was weighed and added to the solution via syringe through a rubber septum. The reaction mixture was maintained under a nitrogen atmosphere with stirring at 60 °C for an additional 2 d. On the third day, the mixture was concentrated using a rotary evaporator at 40–45 °C under vacuum (60 mbar) to achieve a concentration of approximately 20 wt%. To further process the reaction mixture, 34 mL of 1‐methoxy‐2‐propanol was added, and the solvent was subsequently removed again using a rotary evaporator to maintain the 20 wt% concentration. The final product HSNP@PEG@NH_2_ (3) was purified by dialysis in 1‐methoxy‐2‐propanol using a Spectra/Por 7 MWCO 3500 dialysis membrane. The dialysis process was carried out over 3 d, with the solvent being replaced daily to ensure thorough purification.

### Synthesis of HSNP@PEG@NH_2_@FITC (4)

To synthesize HSNP@PEG@NH_2_@FITC (4), a dispersion of HSNP@PEG@NH_2_ (33.5 mmol) in 1‐methoxy‐2‐propanol was mixed with a solution of fluorescein 5(6)‐isothiocyanate (1.305 mg, 3.4 µmol) in ethanol. The reaction mixture was stirred at room temperature for 20 h to achieve functionalization. Following the reaction, the mixture was purified through dialysis in dimethylformamide (DMF) using a Spectra/Por 7 MWCO 3500 dialysis membrane. The dialysis process was conducted over 2 d, with the solvent being replaced daily, yielding a slightly yellowish suspension in DMF. DLS and TEM were used to characterize the resulting HSNP@PEG@NH_2_@FITC nanoparticles.

### Synthesis of HSNP@PEG@NH_2_@FITC@COOH (5)

For HSNP@PEG@NH_2_@FITC@COOH (5) synthesis, a dispersion of HSNP@PEG@NH_2_@FITC (10.7 mmol) in DMF was degassed under a nitrogen atmosphere for 30 min. A solution of succinic anhydride (25.6 mg) in DMF was then slowly added to the mixture. The reaction was stirred at room temperature for 20 h, maintaining the nitrogen atmosphere. Following the reaction, the product was purified by dialysis in deionized water using a Spectra/Por 7 MWCO 3500 dialysis membrane. Dialysis was carried out over 5 d, with the solvent replaced daily, resulting in a slightly yellowish dispersion in water. To ensure complete conversion of amine groups to carboxylic acid groups, an excess of 20% succinic anhydride was used.

### Synthesis of Antibody Conjugated biHSNP (6)

Antibody conjugation onto the surface of HSNPs was achieved using carbodiimide chemistry to activate surface carboxylic acid functionalities. Carboxylic acid groups were reacted with 1‐ethyl‐3‐(3ʹ‐dimethylaminopropyl) carbodiimide (EDC) to form an O‐acylisourea intermediate, which was subsequently stabilized by N‐hydroxysuccinimide (NHS) to generate an NHS ester. This intermediate, more stable than the O‐acylisourea form, enabled efficient conjugation to primary amines under physiologic conditions. In detail, HSNP@PEG@NH_2_@FITC@COOH (3.8 mg, 3.07 × 10^−4^ mol) were activated by incubation with NHS (0.706 mg, 6.14 × 10^−6^ mol) and EDC (1.18 mg, 1.23 × 10^−5^ mol) in MES buffer (pH 6.8) at room temperature for 30 min. Following activation, the nanoparticles were centrifuged and washed twice with deionized water to remove unreacted reagents. The activated HSNPs were then incubated with monoclonal antibodies (400 mg) dissolved in PBS buffer under gentle shaking for 16 h at room temperature. After the reaction, biHSNPs were purified by centrifugation and washed twice with PBS to eliminate unbound antibodies. A total of eight antibody‐conjugated nanoparticles were successfully prepared in this study: HSNP_αCD3_, HSNP_αCD16,_ HSNP_αCD19_, HSNP_αC20_, HSNP_αCD3_^+^_αCD20_, HSNP_αCD3_^+^_αCD19_, HSNP_αCD16_^+^_αCD20_, HSNP_αCD16_^+^_αCD19_.

### Fourier‐Transform Infrared (FT‐IR) Spectroscopy

FT‐IR spectra were recorded using a BRUKER TENSOR 27 FT‐IR spectrometer with the KBr pellet technique. Prior to analysis, samples were dried at 45 °C overnight. Measurements were performed at room temperature, covering a spectral range of 400 to 4000 cm^−1^.

### Dynamic Light Scattering (DLS)

DLS measurements were performed on a Zetasizer Nano ZS (Malvern) using disposable polystyrene cuvettes (DTS0012). A 25 µL aliquot of the dispersion was diluted with 1000 µL of water (HPLC Gradient Grade, Carl Roth) to minimize multiple scattering effects. Hydrodynamic diameter (*D*_h_) and polydispersity index (PDI) were measured at an angle of 173° at 25 °C, following an equilibration period of 60 s. Data analysis was conducted using Zetasizer Software (Malvern).

### Nuclear Magnetic Resonance (NMR) Spectroscopy

Liquid‐state ^1^H NMR spectra were obtained using a Bruker Ascend 400 NMR spectrometer operating at 400 MHz. Measurements were conducted in D_2_O at ambient temperature, with chemical shifts (δ) reported in parts per million (ppm) and referenced to the trace proton signal in D_2_O (δH = 4.79 ppm).

### Transmission Electron Microscope (TEM)

TEM images were captured using a FEI Tecnai G2 F20 field emission scanning electron microscope operating at an accelerating voltage of 200 kV. For sample preparation, a drop of diluted dispersion was deposited onto a carbon‐coated copper grid (Beijing Zhongjingkeyi Films Technology) and air‐dried under ambient conditions for 48 hours.

### Thermogravimetric Analysis (TGA)

TGA was conducted on a METTLER TOLEDO TGA/DSC1 instrument under a nitrogen atmosphere. Samples were heated at a rate of 10 K min^−1^. Particles, isolated via centrifugation and dried at 70 °C overnight, were loaded into aluminum crucibles in quantities of 5–10 mg for analysis.

### Absorption Measurements

Absorption spectra were measured using a Tecan Infinite 200 PRO absorbance microplate reader. Sample volumes of 100 µL were analyzed on a microplate at a wavelength of *λ* = 570 nm, under ambient temperature conditions.

### Ninhydrin Assay

The degree of amino functionalization on silica hollow nanoparticle surfaces was determined via a ninhydrin assay, which relies on the reaction of primary amine groups with ninhydrin in a 2 mol L^−1^ acetic acid buffer (pH 6.0), forming Ruhemann's purple with a characteristic absorption peak. A calibration curve was generated using a series of known concentrations of N^1^‐(3‐trimethoxysilylpropyl)diethylenetriamine. Each solution (100 µL) was reacted with ninhydrin reagent (100 µL) and acetic acid buffer (500 µL) at 80 °C for 15 min while stirring at 400 rpm on an IKA RCT basic magnetic stirrer. Absorbance was measured at λ = 570 nm using a Molecular Devices SpectraMax M2 absorbance microplate reader. Each sample (100 µL) was characterized under identical conditions for comparison.

### Cell Line Culture

The cell lines HEK293T (RRID: CVCL_0063), Raji (CVCL_0511), L428 (CVCL_1361), and KMH2 (CVCL_1330) were purchased from the American Type Culture Collection (ATCC, Manassas, USA). Selected cell lines were genetically modified to stably express firefly luciferase for cellular viability assays described below. All cell lines were cultured in DMEM or RPMI‐1640 medium (Gibco, Carlsbad, CA, USA) supplemented with 10% fetal bovine serum, 100 U mL^−1^ penicillin, and 100 µg mL^−1^ streptomycin (Gibco, Carlsbad, CA, USA). Cell cultures and in vitro assays were conducted in a humidified incubator at 37 °C with 5% CO_2_. Mycoplasma contamination was routinely tested and ruled out using commercial detection kits.

### Plasmids

The pLV[Exp]‐Puro‐EF1A>luciferase plasmid was procured from Gefanbio (Shanghai, China). Additional plasmids, psPAX2 and BaEVRless, were kindly provided by Professor Xin Mu. Plasmid DNA used for transfection was prepared from E. coli XL1‐Blue cells using the Qiagen Maxi Prep kit (Qiagen, Valencia, CA, USA).

### HEK 293T Transfection

HEK293T cells were seeded at 70% confluency and incubated overnight at 37 °C in antibiotic‐free DMEM medium. The following day, the medium was replaced with 4 mL of Opti‐MEM reduced serum medium (Opti‐MEM, Gibco). A transfection mixture was prepared by combining 12 µL of TransIT reagent, 400 µL of Opti‐MEM, 9 µg of pLV[Exp]‐Puro‐EF1A>Luciferase plasmid, 3.9 µg of psPAX2, and 2.1 µg of BaEVRless. This mixture was incubated for 20 min at room temperature before being added dropwise to the cells. After overnight incubation at 37 °C, the medium was replaced with DMEM supplemented with 30% FBS and 1% penicillin–streptomycin. Cell supernatants were collected after 48 h, centrifuged at 100 rpm for 5 min, and filtered through 0.45 µm filters to collect viral particles, which were stored at −80 °C.

### Luciferase Transduction of Tumor Cells

Tumor cells (Raji, KMH2, or L428) were seeded at a density of 500 000 cells mL^−1^ and incubated overnight at 37 °C. On the following day, 500 µL of viral vector containing the luciferase gene was mixed with 8 µg mL^−1^ polybrene, incubated at room temperature for 15 min, and added to the cells. Selection began on day 3 by replacing the culture medium with selection medium containing 3 µg mL^−1^ puromycin. The selection process continued for 10 d, with media changes every 48 h. Cell viability was assessed during each media change to ensure successful transduction.

### Confocal scanning Laser Microscopy

Confocal microscopy was performed on a Leica TCS SP8 system to visualize binding ability of antibody‐conjugated HSNPs to target cells and cell–cell interaction efficiency of Jurkat cells stained with CellTracker Green CMFDA, and Raji cells stained with CellTracker Red CMTPX in the presence or absence of biHSNPs. Co‐cultured cells were placed on a glass substrate, covered with a coverslip, and imaged using lasers matched to the excitation profiles of the dyes. The fluorescence signal was collected by separate detectors to ensure accurate visualization.

### Flow Cytometry Study of biHSNP‐Cell Interaction

The binding ability of antibody‐conjugated HSNPs to target cells was evaluated via flow cytometry. Target cells (1 × 10⁶) were suspended in 300 µL PBS (1×) and incubated with 500 µL of 2% BSA at 4 °C for 1 h to block nonspecific binding. The cells were centrifuged at 3000 rpm for 5 min, and the supernatant was discarded. Nanoparticle dispersions were added, followed by incubation in the dark for 1 h. Unbound particles were removed by two rounds of washing with PBS. Cell suspensions were analyzed using a BD FACSVerse flow cytometer, and data were processed with FlowJo software.

### Flow Cytometry Study of Cell–Cell Interaction Efficiency

Jurkat cells were stained with CellTracker Green CMFDA, and Raji cells were stained with CellTracker Red CMTPX. Stained cells were washed twice with PBS and suspended in binding buffer at 4 °C. Jurkat cells (1 × 10⁶) were incubated with 20 µL (2 mg mL^−1^) biHSNPs in binding buffer for 30 min at 4 °C. Subsequently, Raji cells were added at a 1:1 ratio and co‐incubated for 1 h at 4 °C. The cell–cell interactions were analyzed using a BD FACS Calibur flow cytometer.

### Cytotoxicity Assays

Cytotoxicity assays were conducted to assess the ability of biHSNPs to enhance immune cell‐mediated cytotoxicity against malignant cells. Luciferase‐transduced target cells (Raji_luc, KMH2_luc, L428_luc) were seeded at 10⁴ cells per well in a 96‐well plate. PBMCs were added at an effector‐to‐target cell ratio of 20:1, followed by treatment with various concentrations of nanoparticle dispersions. Cell viability was monitored over time using bioluminescence measurements. Luciferin (Regis Biotechnology, Morton Grove, IL, USA) was added to each well at a final concentration of 200 µg mL^−1^, and luminescence was measured 48 hours later using a SpectraMax i3 plate reader (Molecular Devices, San Jose, CA). Luminescence signals, directly proportional to viable cells, were analyzed to evaluate cytotoxic effects. Control wells without nanoparticles and blank wells were included in all experiments. Each measurement was performed in triplicate.

### Enzyme‐Linked Immunosorbent Assay (ELISA)

ELISA tests for human IL‐2, TNF‐α, and IFN‐γ were performed following the ELISA MAX Deluxe Set (Biolegend) protocol. Briefly, 96‐well plates were coated with 100 µL of diluted capture antibody solution and incubated overnight at 2–8 °C. After washing the plate four times with Wash Buffer and blotting residual buffer, wells were blocked with 200 µL of 1X Assay Diluent A to prevent non‐specific binding, followed by a 1 h incubation at room temperature (RT) with shaking. Standards and samples were prepared during the blocking step. After washing, 100 µL of standards or diluted samples (25 µL sample ^+^ 75 µL 1X Assay Diluent A) were added to the wells and incubated for 2 h at RT with shaking. Wells were then washed, and 100 µL of diluted detection antibody solution was added, followed by a 1 h incubation at RT with shaking. After washing again, 100 µL of diluted Avidin‐HRP solution was added and incubated for 30 min at RT with shaking. Following a final wash (five cycles, with 30 s to 1 min of soaking per cycle), 100 µL of TMB substrate solution D was added to each well and incubated in the dark for 15 min. The reaction was stopped by adding 100 µL of stop solution. Absorbance was measured at 450 nm and 570 nm within 15 min, with values at 570 nm subtracted from those at 450 nm to eliminate background noise. Cytokine levels (IL‐2, TNF‐α, and IFN‐γ) were calculated by comparing sample absorbance to a standard curve. Results were normalized to control samples and reported as fold increases.

### Mice

Nu mice were obtained from SpePharm (Beijing) Biotechnology Co., Ltd. All animals were housed and experiments conducted in a sterile environment. Mice were provided ad libitum access to food and water and were monitored daily for health. Cages were cleaned and replaced weekly.

### Nude Mouse Xenograft Model

Six‐week‐old female BALB/c nude mice were purchased from SpePharm (Beijing) Biotechnology Co., Ltd., and randomly assigned to seven groups (*n* = 6 per group). Approximately 1 × 10⁷ Raji cells were resuspended and injected subcutaneously into the flanks of each mouse. Peripheral blood T cells were isolated from healthy human donors using a human Pan T Cell Isolation Kit (Xinbio, Suzhou, China). Two weeks after Raji cell inoculation, 5 × 10⁶ T cells were administered intravenously to each mouse weekly. In a preliminary study to determine the optimal dose of biHSNPs, mice were treated with HSNPαCD3^+^αCD19 at 1, 2, or 4 mg kg^−1^. Based on these findings, a dose of 2 mg kg^−1^ was selected for subsequent experiments. One hour after T cell injection, mice received intravenous treatments with bispecific nanoparticles (HSNP_αCD3_, HSNP_αCD19_, HSNP_αCD20_, HSNP_αCD3_^+^_αCD19_, or HSNP_αCD3_^+^_αCD20_) at 2 mg kg^−1^, administered three times per week. Tumor length (*D*), width (*d*), and body weight were measured every 3 d. At 29 d post‐inoculation, mice were sacrificed, and xenografts were excised, weighed, and analyzed. Tumor volumes were calculated using the formula: volume = (*D*×*d* ^2^)/2. Xenografts were further subjected to immunohistochemistry (IHC), immunofluorescence (IF), and flow cytometry analyses to evaluate protein expression and tumor characteristics.

### Immunohistochemistry (IHC)

Formalin‐fixed tumor tissues were embedded in paraffin and sectioned into 5 µm slices, which were mounted on glass slides. After deparaffinization and rehydration, antigen retrieval was performed. The sections were incubated overnight at 4 °C with primary antibodies against CD8 (1:500, cat. no. OM641943, OmnimAbs, USA) and CD19 (1:500, cat. no. OM641833, OmnimAbs, USA). Subsequently, the slides were treated with secondary antibodies for 2 h at room temperature. Pathological images were captured, and optical density was quantitatively analyzed using Image‐Pro Plus (IPP) 6.0 software (Media Cybernetics Inc., USA).

### Immunofluorescence Analysis (IF)

Tissue sections were fixed in 4% paraformaldehyde for 15 min and permeabilized with 0.2% Triton X‐100 for 5 min. The slides were then incubated overnight at 4 °C with primary antibodies against CD8 (1:500, cat. no. OM641943, OmnimAbs, USA) and CD19 (1:500, cat. no. OM641833, OmnimAbs, USA). Afterward, a secondary antibody conjugated to a green fluorophore was applied in the dark. Nuclear staining was performed with DAPI for 5 min at room temperature. Images were captured using a laser‐scanning confocal microscope (LSM‐710, Zeiss, Germany).

### Statistics and Reproducibility

All experiments were independently performed in triplicate. Data were presented as mean ± standard deviation (SD). Continuous variables were expressed as mean ± SD or median and interquartile range, and comparisons between two groups were accomplished using the Student's t‐test. One‐way ANOVA was used to compare the mean between three or more subgroups. A Spearman correlation was conducted to explore the association between the two variables. The statistical significances are indicated separately in each individual graph.

## Conflict of Interest

The authors declare no conflict of interest.

## Author Contributions

Y.S. and X. L. contributed equally to this work. Y.S. data curation, formal analysis, investigation, and writing—original draft. X.L. conceptualization, investigation, supervision, writing—review & editing. J.W. investigation. Y.M. visualization. S.B. investigation. Z.C. formal analysis. Y.W. data curation. Y.Z. investigation. J.S. writing—review & editing. X.L. conceptualization, funding acquisition, writing—review & editing. Y.T. conceptualization, writing—review & editing. Z.S. conceptualization, funding acquisition, project administration, supervision, visualization, and writing—review & editing.

## Supporting information



Supporting Information

## Data Availability

The data that support the findings of this study are available in the Supporting Information of this article.
