# Supplementary material for: Bispecific Nanosystems Enable Multieffector Immune Cell Retargeting for Hematologic Malignancy Therapy
Source: Adv Sci (Weinh). 2025 Aug 11;12(39):e09103. doi: 10.1002/advs.202509103 (PMC12533383; doi:10.1002/advs.202509103)
Supplement: Supplementary file 1 — Supporting Information [file ADVS-12-e09103-s001.docx]

**Supporting Information**

for

**Bispecific nanosystems enable multi-effector immune cell retargeting for hematologic malignancy therapy**

*Yefeng Shen^1,2^*^†^*, Xin Li*^3†^**, Jingnan Wu^4,5^, Yuru Ma^1^, Sven Borchmann^6^, Zhenguo Cheng^7^, Yaohe Wang^8^, Yongliang Zhao^9^, Jian Song^10^, Boyu Luo^4^, Xiuyun Liu^1,11^*, Yue Teng^4^*, Zhiyuan Shi^1,6^*.*

^1^School of Pharmaceutical Science and Technology, Faculty of Medicine, Tianjin University, Tianjin, 300072, China

^2^Department of Thoracic Surgery, Beijing Friendship Hospital, Capital Medical University, Beijing, 100050, China

^3^Department of Biomedical Engineering, City University of Hong Kong, Hong Kong, 999077, China

^4^State Key Laboratory of Pathogen and Biosecurity, Beijing Institute of Microbiology and Epidemiology, Beijing, 100071, China

^5^Institute of Technical and Macromolecular Chemistry, RWTH Aachen University, Aachen, 52074, Germany

^6^Department I of Internal Medicine, University of Cologne, Köln, 50937, Germany

^7^National Centre for International Research in Cell and Gene Therapy, Sino-British Research Centre for Molecular Oncology, State Key Laboratory of Esophageal Cancer Prevention & Treatment, School of Basic Medical Sciences, Academy of Medical Sciences, Zhengzhou University, Zhengzhou, 450001, China

^8^Centre for Cancer Biomarkers & Biotherapeutics, Barts Cancer Institute, Queen Mary University of London, London, United Kingdom

^9^Ningbo Dilato Materials Co., LTD, 581 South Zhuangyu Road, Zhenhai District, Ningbo, 315200, China

^10^Institute of Cardiovascular Sciences, Guangxi Academy of Medical Sciences, Nanning, 530021, China

^11^Neurocritical Care Medicine Innovation Center, Ministry of Education, Tianjin University, Tianjin, 300072, China

*Correspondence to: zhiyuan_shi2023@tju.edu.cn (Z. Shi); xli@dwi.rwth-aachen.de (X. Li); yueteng@sklpb.org (Y. Teng); xiuyun_liu@tju.edu.cn (X. Liu)

**Table of content**

[1 Additional synthetic procedures and characterization data 3](#_Toc185344361)

[1.1 Additional characterization data of HSNP@PEG@NH_2_ 4](#_Toc185344362)

[1.2 Determination of amino functionalization on HSNP@PEG@NH_2_ via ninhydrin assay 6](#_Toc185344363)

[1.3 Characterization of HSNP@PEG@NH_2_@FITC 8](#_Toc185344364)

[1.4 Characterization of HSNP@PEG@NH_2_@FITC@COOH 8](#_Toc185344365)

[2 Determination of concentration of antibody coated on the surface of HSNPs 10](#_Toc185344366)

[3 Additional FACS and confocal microscopy results 12](#_Toc185344367)

[4 Additional cytotoxicity of HSNPs 15](#_Toc185344368)

[5 Preliminary in vivo tumor growth inhibition experiments 19](#_Toc185344369)

# Additional synthetic procedures and characterization data


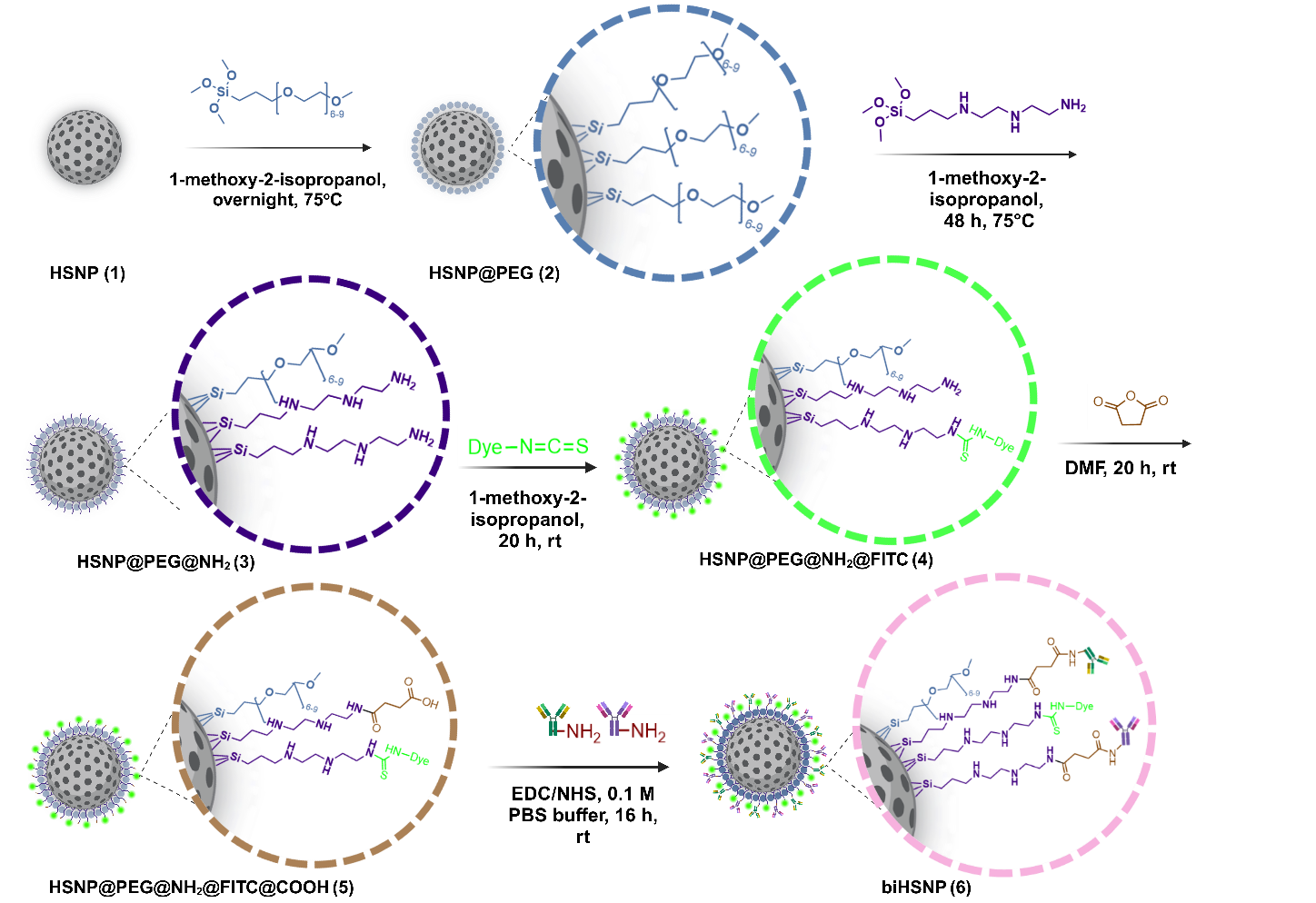


Scheme S1. Synthetic route of biHSNPs.

## Additional characterization data of HSNP@PEG@NH_2_

The Fourier transform-infrared spectroscopy (FT-IR) spectra of HSNP@PEG, along with unmodified HSNP for comparison, are shown in Figure 1, B. Additional bands at 2922.1 cm⁻¹, corresponding to C-H bending vibrations characteristic of HSNP@PEG, confirm the successful surface modification of HSNP with PEG. The characteristic stretching bands of HSNP@PEG’s C-O-C bond in the 1300–1000 cm⁻¹ range were not observed, likely due to masking by the dominant silica nanosphere signals. Peaks at 3419.8 cm⁻¹ (–OH stretching), 1099.4 cm⁻¹ (asymmetric Si-O-Si stretching), 800.7 cm⁻¹ and 468.7 cm⁻¹ (symmetric Si-O-Si stretching), and 956.7 cm⁻¹ (Si-OH stretching) were evident. A water-related peak was detected at 1640 cm⁻¹.

The ¹H NMR spectrum further validated the successful HSNP@PEG, as evidenced by the characteristic proton peaks at δ = 3.70 ppm (–OCH₂CH₂O–) and δ = 3.39 ppm (CH₃O–) (Figure 1, C and Figure S1, A).

Dynamic light scattering (DLS) and transmission electron microscopy (TEM) were used to further characterize HSNP and HSNP@PEG (Figure 1, D). DLS analysis revealed an increase in the hydrodynamic diameter (Dₕ) upon PEG substitution, with a relatively low polydispersity index (PDI) of 0.154, indicating uniform particle size distribution. A summary of the particle size and related data is provided in Table S2.


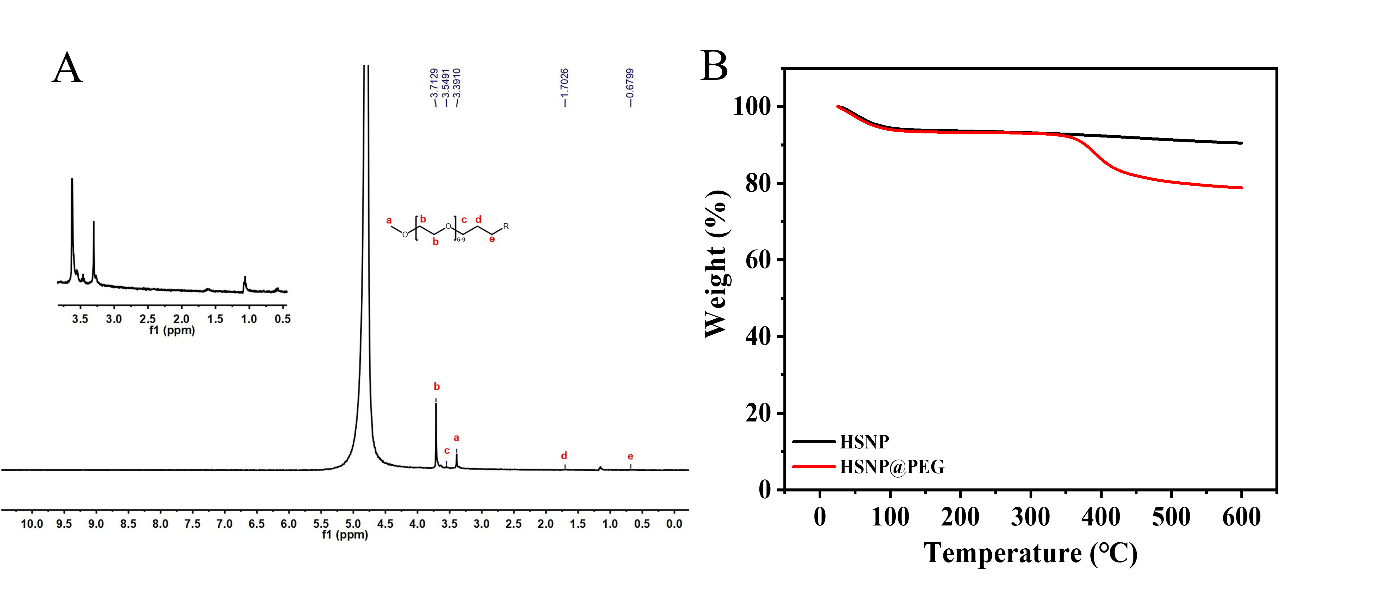
Figure S1. A) ^1^H NMR spectra of HSNP@PEG in D_2_O. B) TGA curves of HSNP and HSNP@PEG.

Thermogravimetric analysis (TGA) was performed to assess the degree of PEGylation. TGA curves were recorded under a nitrogen atmosphere from 20 to 600 °C, with the residual mass percentage of each sample measured. Figure S1, B illustrates the TGA curves for HSNP and HSNP@PEG. Unmodified HSNP exhibited a slight, continuous mass loss between 20 and 100 °C, attributed to the removal of adsorbed water and silanol condensation. In contrast, HSNP@PEG displayed significant mass loss between 350 and 600 °C, corresponding to the decomposition of PEG groups.

The degree of PEGylation was quantified using the equation:

$$n\left( PEG\% \right)=\frac{m_{\mathrm{res}}\left( a \right)-m_{\mathrm{res}}\left( i \right)}{m_{\mathrm{res}}\left( a \right)}$$

Here, m_res_(a) represents the residual mass of unmodified HSNP after accounting for water loss at 100 °C, and m_res_(i) represents the residual mass of HSNP@PEG after calcination at 600 °C. The calculated PEG mass fraction was 16.0% (Table S1). The theoretical PEG mass fraction was also estimated based on the masses of the reactants.

Table S1**.** Summary of PEG wt.-% obtained by TGA measurements and from the reaction procedure.

| Sample | TGA PEG content  *wt.-*% [%] | Theoretical PEG content  *wt.-*% [%] |
| --- | --- | --- |
| HSNP@PEG | 16.0 | 42 |

## Determination of amino functionalization on HSNP@PEG@NH_2_ via ninhydrin assay

The degree of amino functionalization on the surface of HSNP@PEG@NH2 was quantified using the Ninhydrin assay (Figure S2, A). A standard solution of 3-[2-(2-Aminoethylamino)ethylamino]propyl-trimethoxysilane was prepared in HAC and AC-buffered solution (pH 6.0) at a concentration of 10.00 mM. Serial dilutions ranging from 0.025 mM to 0.500 mM were reacted with ninhydrin at 80 °C for 15 minutes, producing Ruhemann’s purple (Figure S2, B). UV absorbance of the reaction mixtures was measured at full wavelengths using a microplate reader, with the maximum absorption observed at 566 nm (Figure S2, C). A standard curve of absorbance at 566 nm versus concentration was constructed, yielding a linear regression equation: y = 2.92x − 0.03 (Figure S2, D).

To determine the amino functionalization on HSNP@PEG@NH_2_, 1 mL of the nanoparticle dispersion was reacted with ninhydrin under identical conditions. The absorbance of the resulting solution at 566 nm was measured as 0.7199 (Figure S2, E). Substituting this value into the standard curve equation provided a calculated concentration of 0.257 mM. Using this concentration, the degree of amino functionalization was calculated as 15.75 μmol/g in HSNPs.


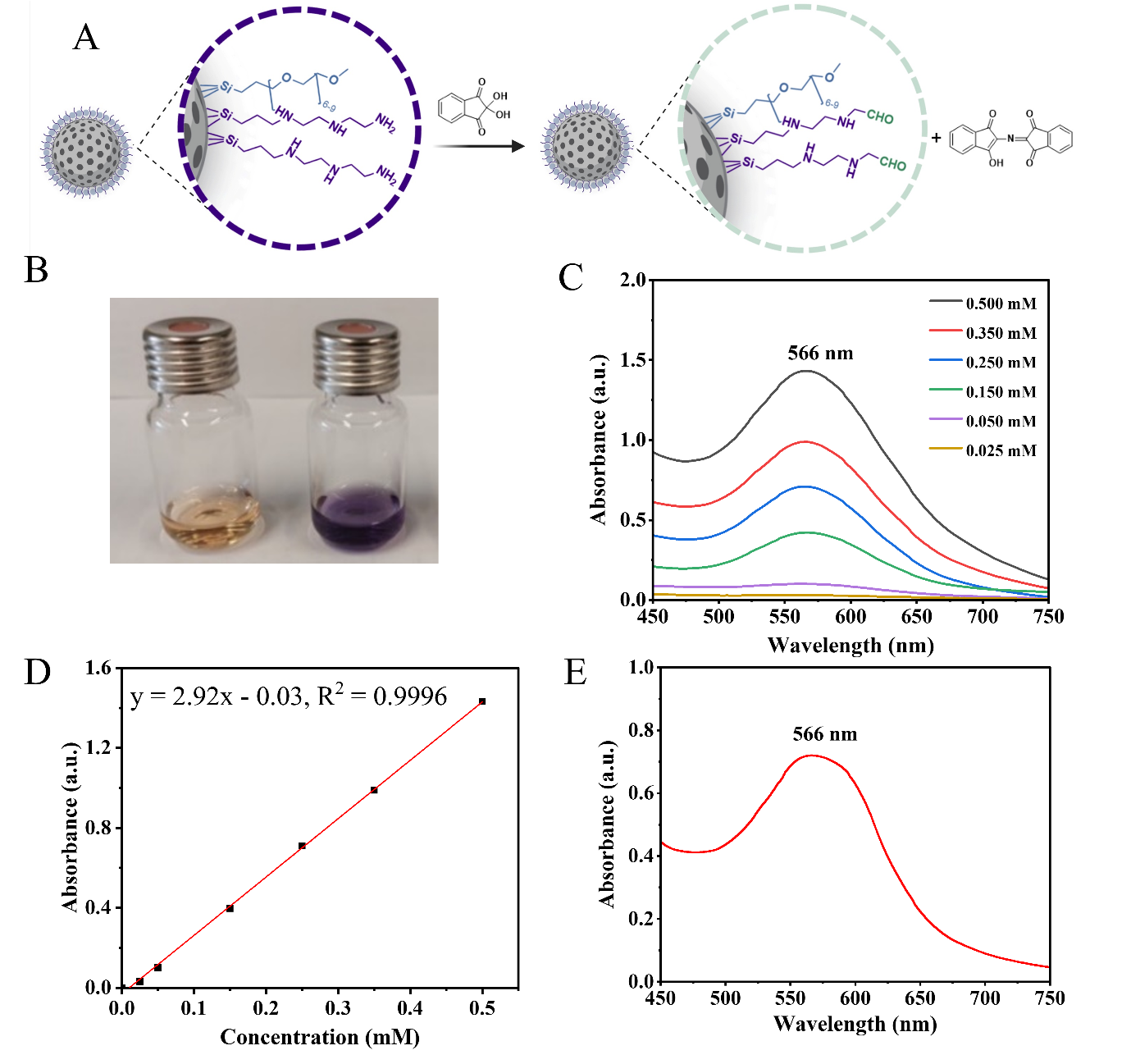
Figure S2**.** A) Schematic illustration of the formation of Ruhemann's purple via reaction of ninhydrin with HSNP@PEG@NH_2_. B) Photos of the reaction mixtures obtained by mixing ninhydrin solution and HSNP@PEG (dark yellow) in comparison to ninhydrin solution and HSNP@PEG@NH_2_ (purple)， The formation of Ruhemann's purple confirmed the successful amino functionalization of HSNP@PEG@NH_2_. C) UV-vis spectra by using different concentrated 3-[2-(2-Aminoethylamino)ethylamino]propyl-trimethoxysilane as the amino source for the ninhydrin assay. D) Standard curve of Aminoethylamino)ethylamino]propyl-trimethoxysilane as the amino source from the ninhydrin assay at 566 nm. E) UV-vis spectra of synthesized HSNP@PEG@NH_2_ for the ninhydrin assay.

## Characterization of HSNP@PEG@NH_2_@FITC

The degree of FITC functionalization was quantified through fluorescence spectroscopy. FITC solutions with concentrations of 4.00, 3.50, 3.00, 2.50, and 1.25 μM were scanned to determine their fluorescence spectra using a TECAN Infinite 200 PRO plate reader. Measurements were conducted in a black, opaque 96-well plate with 1-methoxy-2-propanol serving as the blank control. The maximum fluorescence emission was observed at 522 nm. A standard curve correlating fluorescence intensity to concentration was generated, yielding the equation y = 130.21x - 5.24. The fluorescence spectrum of the HSNP@PEG@NH2@FITC sample was measured, with the maximum fluorescence intensity at 522 nm recorded as 308 (Figure S3). Substituting this value into the standard curve equation yielded an FITC concentration of c = 9.624 μM was obtained and the FITC substitution in HSNP is calculated to be 0.125 μmol/g.


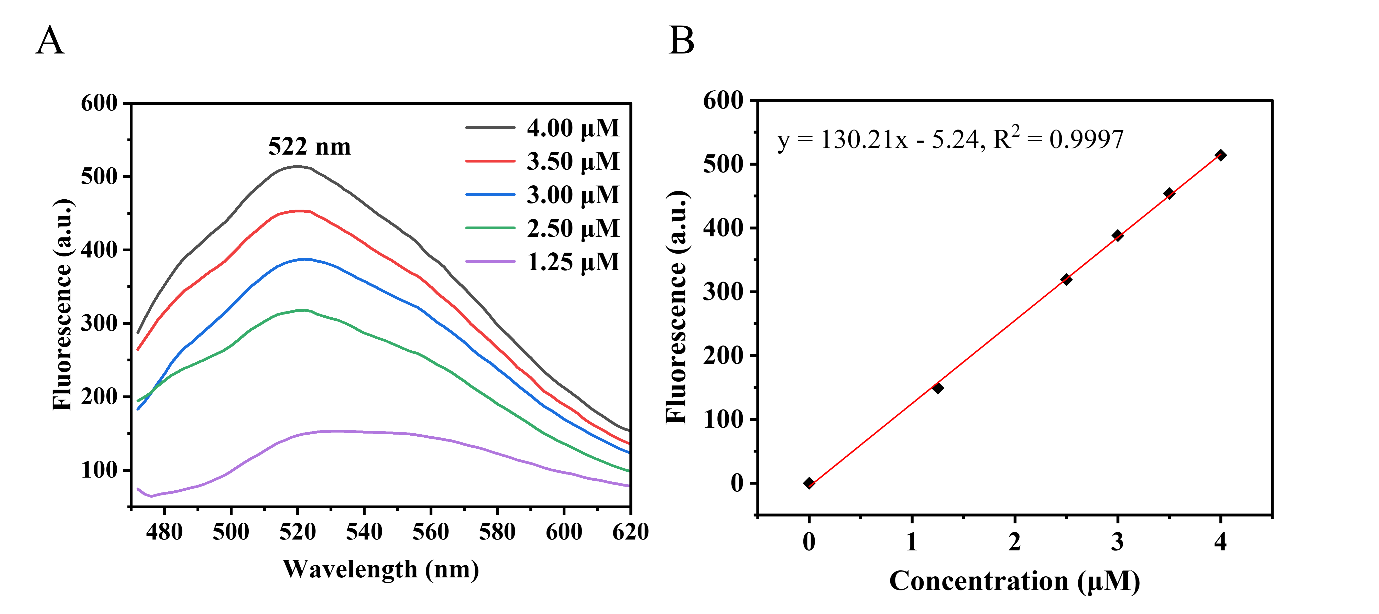
Figure S3. Fluorescence spectra of HSNP@PEG@NH_2_@FITC.

## Characterization of HSNP@PEG@NH_2_@FITC@COOH

The presence of carboxylic acid groups on HSNP@PEG@NH2@FITC@COOH was confirmed by FT-IR analysis. A distinct absorption band at 1701.2 cm^−1^ (Figure S4, A) indicated successful grafting of -COOH functionalities onto the nanoparticle surface. Dynamic light scattering (DLS) measurements determined the hydrodynamic diameter (Dh) of HSNP@PEG@NH2@FITC@COOH as 81.53 nm, with a polydispersity index (PDI) of 0.103 (Figure S4, B). The narrow PDI highlights the uniformity of the modified nanospheres. Furthermore, the particles demonstrated excellent redispersion in water after drying, ensuring stability and retention of desired properties. A summary of the DLS measurements is provided in Table S2.


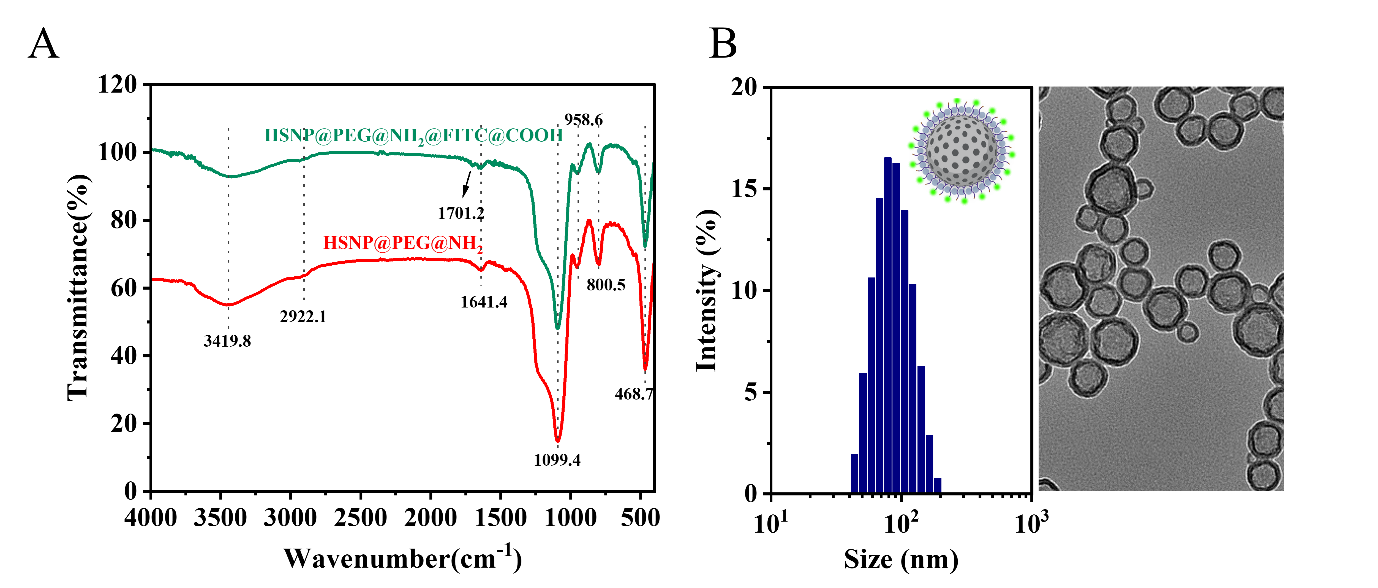


Figure S4**.** (A) FT-IR spectra obtained by HSNP@PEG@NH_2_ (red line) in comparison to HSNP@PEG@NH_2_@FITC@COOH (green line). (B) DLS result and TEM image of HSNP@PEG@NH_2_@FITC@COOH.

Table S2. DLS size measurements of HSNPs in water.

| Sample | D_h_  [nm] | PDI  [ ] |
| --- | --- | --- |
| **HSNP** | **74.64** | **0.159** |
| **HSNP@PEG** | **78.91** | **0.154** |
| **HSNP@PEG@NH_2_** | **90.46** | **0.107** |
| **HSNP@PEG@NH_2_@FITC** | **106.8** | **0.213** |
| **HSNP@PEG@NH_2_@FITC@COOH** | **81.53** | **0.103** |
| **HSNP_αCD3_** | **92.0** | **0.069** |
| **HSNP_αCD16_** | **91.4** | **0.050** |
| **HSNP_αCD19_** | **94.9** | **0.112** |
| **HSNP_αCD20_** | **93.1** | **0.068** |
| **HSNP_αCD3+αCD20_** | **90.3** | **0.033** |
| **HSNP_αCD3+αCD19_** | **92.1** | **0.037** |
| **HSNP_αCD16+αCD20_** | **92.5** | **0.021** |
| **HSNP_αCD16+αCD19_** | **90.7** | **0.032** |

# Determination of concentration of antibody coated on the surface of HSNPs

The concentration of antibodies coated on the surface of biHSNPs was determined using a method analogous to ELISA (Figure S5A). Specifically, PE-conjugated anti-human IgG Fc antibody (BioLegend, 410708) was incubated overnight with HSNPαCD3+αCD20 and HSNPαCD3+αCD19. Following incubation, the nanoparticles were centrifuged at 15,000–18,000 rpm for 20–25 minutes, washed twice with PBS, and resuspended in 100 µL of PBS. The fluorescence intensity of PE on the nanoparticles was then measured.

To quantify the amount of surface-bound antibody, the PE fluorescence intensity of the samples was compared to a standard curve generated using known concentrations of the PE-anti-Fc antibody (Figure S5B). Based on this analysis, the antibody concentration on HSNPαCD3+αCD20 was determined to be 4.08 µg/mL (relative fluorescence intensity = 2610.17), and 4.81 µg/mL for HSNPαCD3+αCD19 (relative fluorescence intensity = 3082.31). Given that the biHSNP concentration used was 1 mg/mL, the final surface antibody densities were calculated as 4.08 µg/mg for HSNPαCD3+αCD20 and 4.81 µg/mg for HSNPαCD3+αCD19.


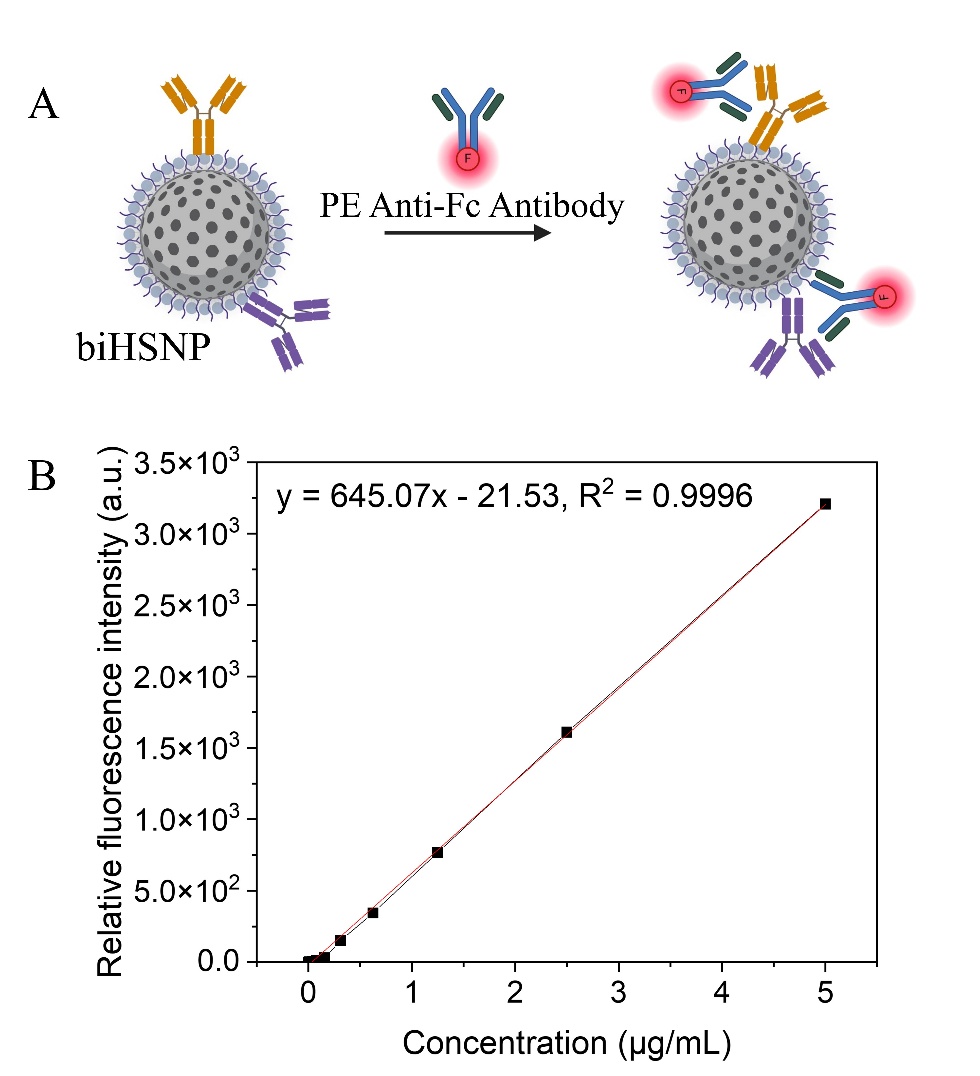


Figure S5. A) Evaluation of antibody coating efficiency on biHSNPs using a fluorophore-labeled secondary antibody targeting the Fc region of the surface-bound monoclonal antibodies. Fluorescence intensity was measured to quantify coating efficiency. B) Standard curve of PE-conjugated human anti-Fc antibody.

# Additional FACS and confocal microscopy results


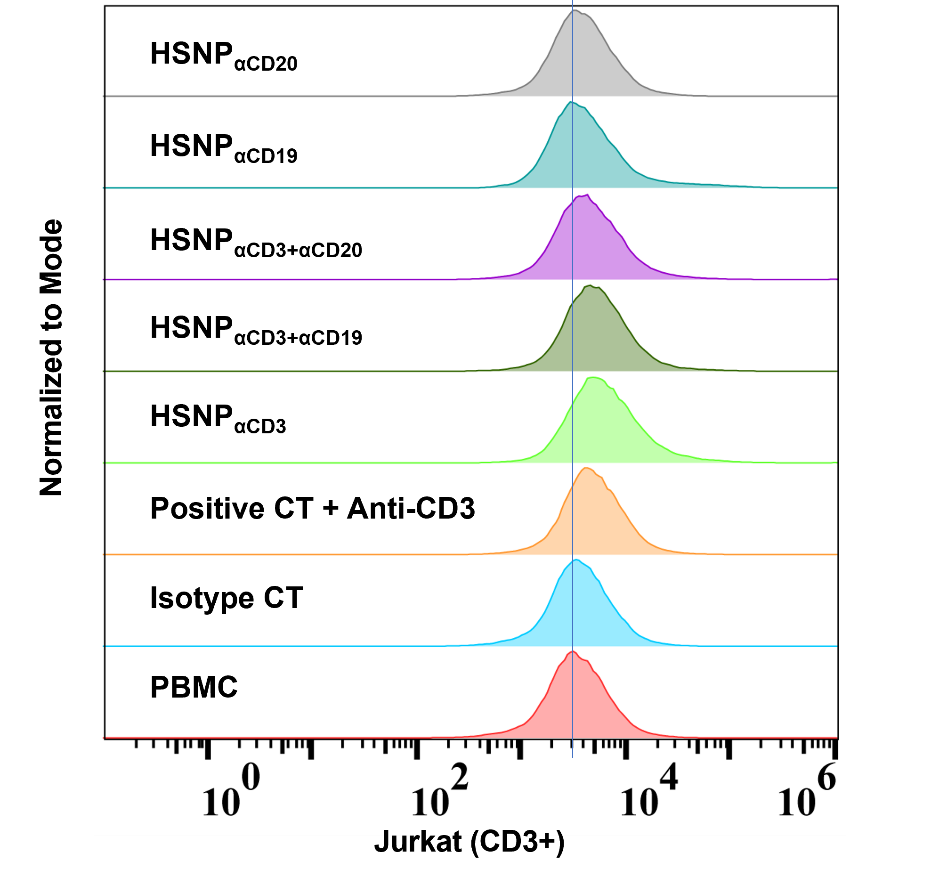


Figure S6. FACS binding results of HSNP_αCD3_, HSNP_αCD19_, HSNP_αCD20_, HSNP_αCD3+αCD19_ and HSNP_αCD3+αCD20_ with Jurkat cells as CD3+ cell lines.


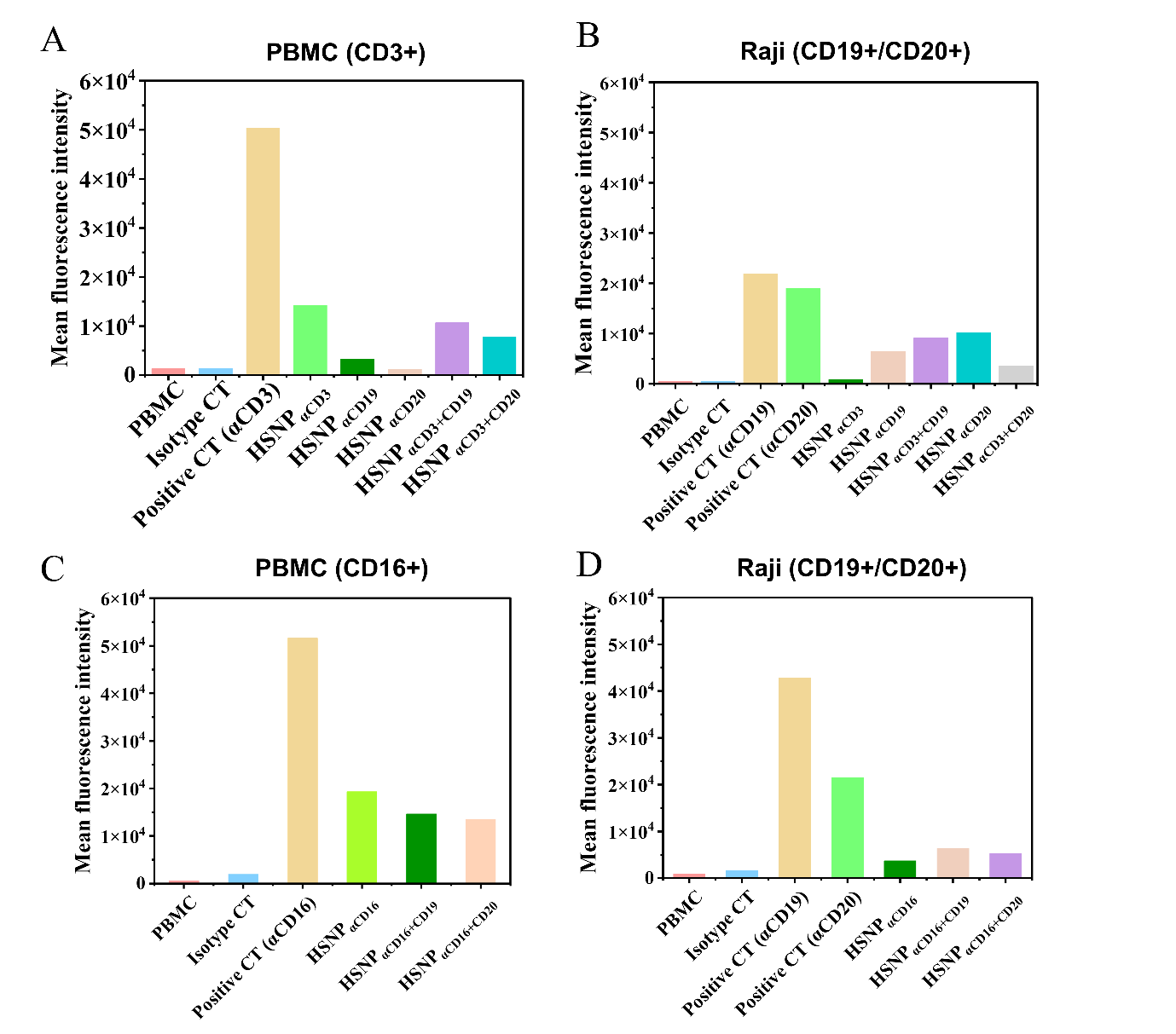
Figure S7. FACS mean fluorescence intensity of antibody conjugated HSNPs with the different cell lines.


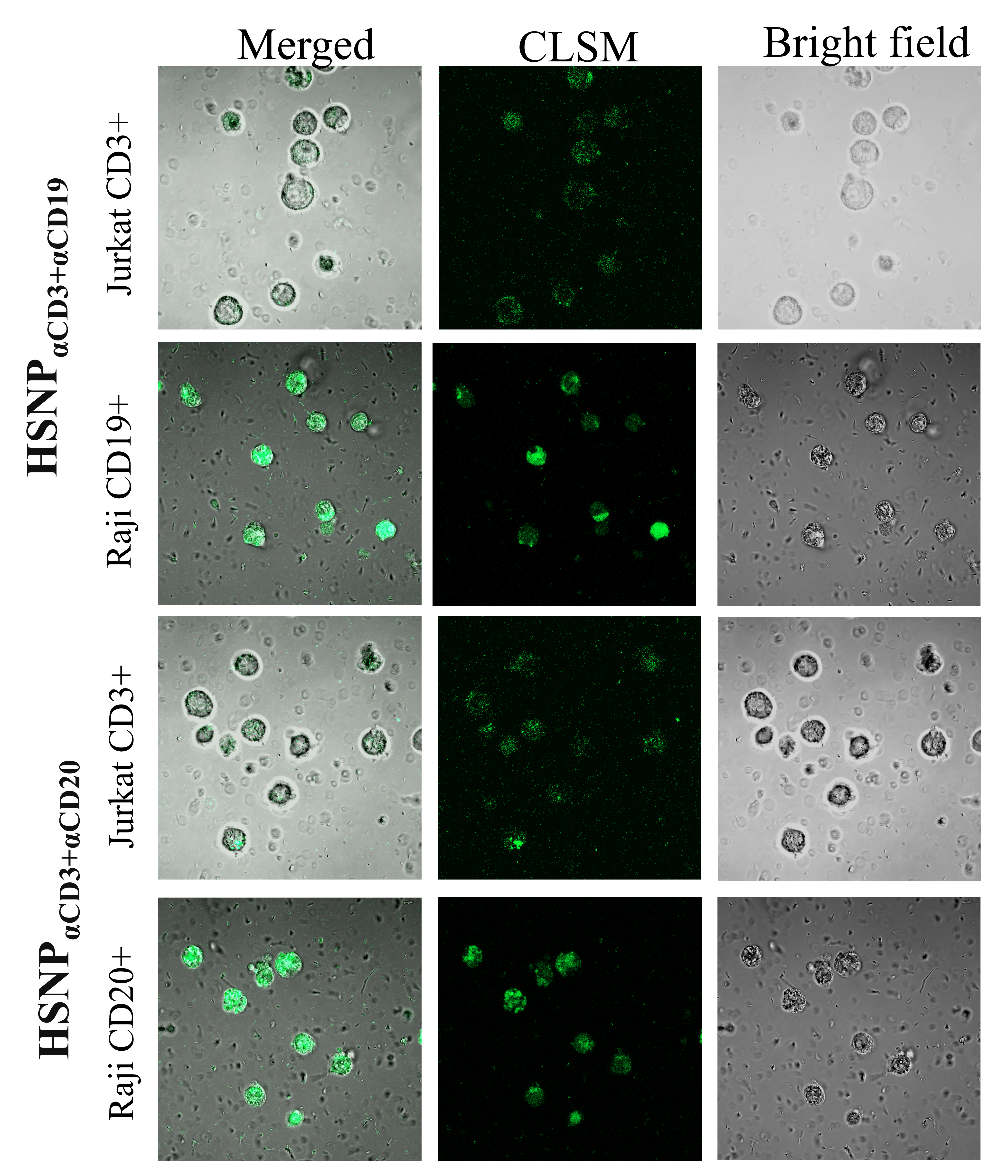


Figure S8. Confocal microscopy images of HSNP_αCD3+αCD19_ and HSNP_αCD3+αCD20_ incubated with either Jurkat as CD3+ or Raji as CD19+/CD20+ positive cell lines.


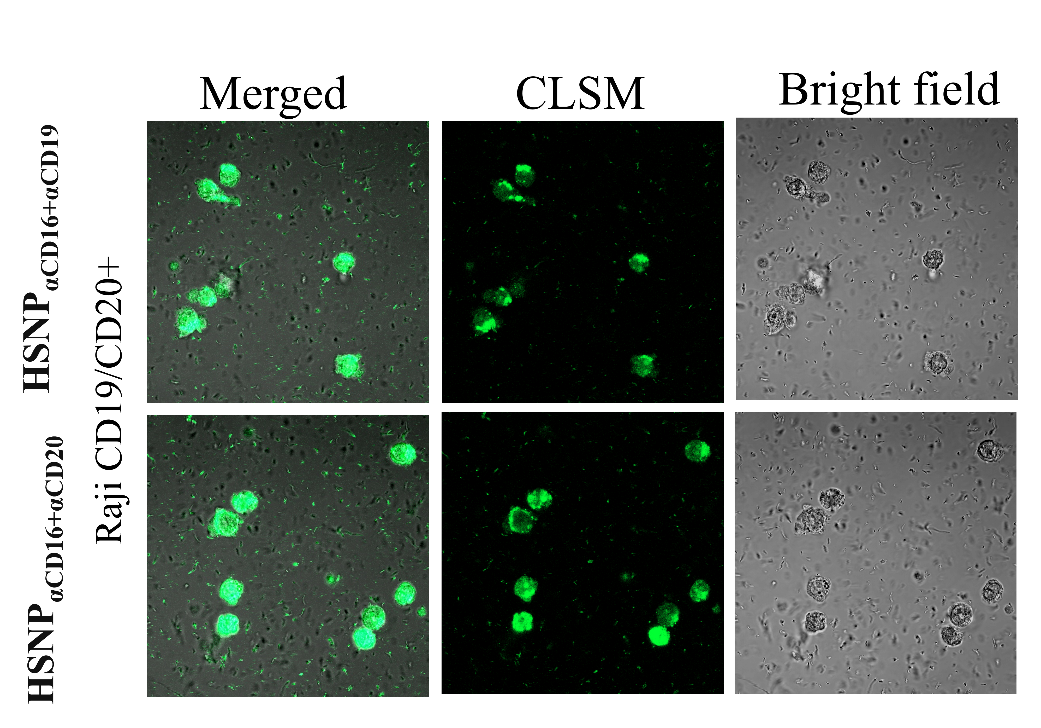


Figure S9. Confocal microscopy images of HSNP_αCD16+αCD19_ and HSNP_αCD16+αCD20_ incubated with Raji as CD19+/CD20+ positive cell lines.

# Additional cytotoxicity of HSNPs

KMH2_luc cell lines were subjected to incubation with various concentrations of nanoparticles (HSNPs) for varying durations. The luminescence intensity was quantified, and cell viability was determined by considering the cells incubated without HSNPs as 100%. Notably, an inverse correlation between HSNPs concentration and cell viability was observed, indicating a decrease in cell viability with increasing HSNPs concentration. Intriguingly, when exposed to HSNPs concentrations exceeding 25 μg/mL, the cells exhibited severe cytotoxicity, leading to cell death. However, when the cell viability assessments with HSNP@PEG, ranging in concentrations from 0 µg/mL to 1000 µg/mL demonstrates minimal cytotoxicity for concentrations up to 100 µg/mL after 72 hours, as the cell viability consistently remains above 100% post-incubation. Substantial toxicity of these particles is only observed at high particle concentrations exceeding 200 µg/mL and after prolonged incubation, indicating significantly reduced toxicity compared to the previously discussed unmodified silica nanospheres (Figure S10). The introduction of PEG modification to the nanoparticles greatly enhances cell viability under identical conditions.


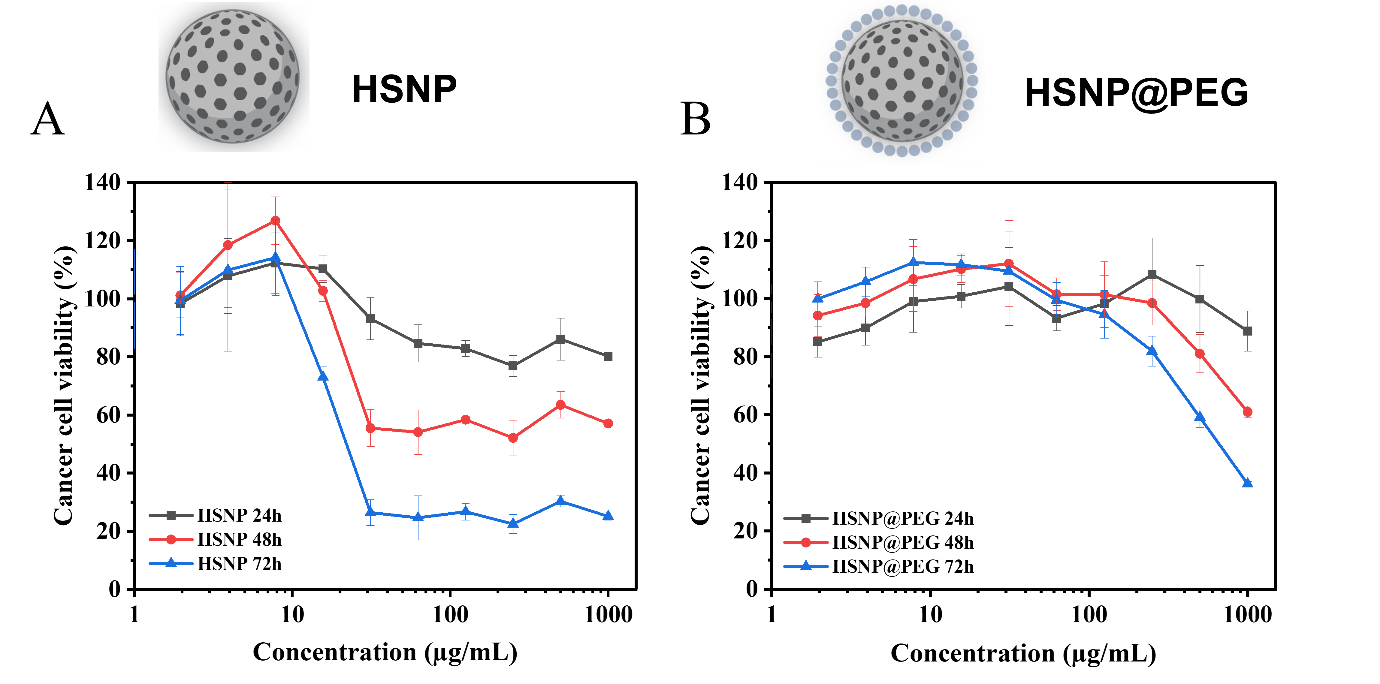


Figure S10. illustrates the cell viability of KMH2 Hodgkin lymphoma cells, as assessed by luminescence measurements, following 24-hour, 48-hour, and 72-hour incubation with HSNP or HSNP@PEG.

We also tested the toxicity of pure antibody using the same experiment condition as the monoHSNPs (Figure S11), our results reveal that pure antibody such as Anti-CD3 antibody(αCD3) and Anti-CD16 antibody(αCD16) show reduced cell viability to ca. 80% at the concentration of 100 ng/mL, similar as the monoHSNP. While co-cultured cells incubated with Anti-CD19 antibody(αCD19) or Anti-CD20 antibody(αCD20) have a cell viability of ca. 60% and 20%, respectively, indicate that both αCD19 and αCD20 can lead to cancer cell death, and αCD20 show higher cell toxicity as the αCD19 under our experiment condition.

Figure S11. Additional toxic assay results of pure antibodies used for the conjugation.

Furthermore, to test specificity of the biHSNP, luciferase transfected human Hodgkin Lyphoma L-428/KMH2 cell line which is a CD19 and CD20 negative cell lines were was utilized as control (Figure S12 and Figure S13). We could observe that the HSNP@PEG@NH2@FITC@COOH without antibody and monoHSNP coated with only αCD19 or αCD20 show neglectable toxicity, while monoHSNP coated with αCD3 we could observe a reduced cell viability to ca. 70% at concentration above 100 μg/mL. Notably, biHSNP coated with both αCD3 and αCD19 or αCD20 show similar toxicity as the monoHSNP coated with only αCD3, which is much lower toxicity as the biHSNP incubated with Raji cells, which implies the specific toxicity to selected cell lines.

Figure S12. Control toxic assay of HSNP@PEG, HSNP_αCD3_, HSNP_αCD19_, HSNP_αCD20_, HSNP_αCD3+αCD19_ and HSNP_αCD3+αCD20_ with L-428_luc (CD19/CD20- negative) cell lines.

Figure S13. Control toxic assay with HSNP_αCD16_, HSNP_αCD16+αCD19_, HSNP_αCD16+αCD20_ with KMH2_luc (CD19/CD20- negative) cell lines.

Figure S14. Additional toxic assay of FITC.

# Preliminary in vivo tumor growth inhibition experiments

Finally, we investigated the efficacy of biHSNP treatment in in vivo models. For the in vivo model, based on our in vitro system, was an adoptive transfer of the repetitively stimulated T cells into mice with pre-established Raji lymphoma tumors, followed by intravenous administration of biHSNP. Adoptively transferred cells were used as a model of exhausted tumor-specific cells that would likely be present in a patient’s tumor microenvironment prior to treatment (Figure S15).

Nude mice were adaptively raised for 1 week, followed by subcutaneous injection of Raji lymphoma cells/Matrigel (100 μL, 1×10^7^ per mouse). Tumor formation was observed after approximately 1 week. Subsequently, interventions were performed based on groups for 3 weeks (intravenous injection of nanoparticles and T cells). biHSNPs injections were administered 3 times per week for 3 weeks, while T cell injections were administered once per week. Moreover, both the body weight and tumor diameter were measured once before intervention and once every 3 days after intervention.

There were no significant differences in tumor volume among the groups before administration (Day 0). After 21 days of administration, the tumor volumes in the T cell group (p = 0.0150) and the low-dose nanoparticle group (p = 0.0019) were significantly lower compared to the model group. After 21 days of administration, there were no significant differences in tumor volume between the low-dose nanoparticle group and the T cell group (p = 0.8831). The tumor volumes in the medium-dose group (p = 0.0383) and high-dose group (p = 0.0206) were significantly smaller than the T cell group.


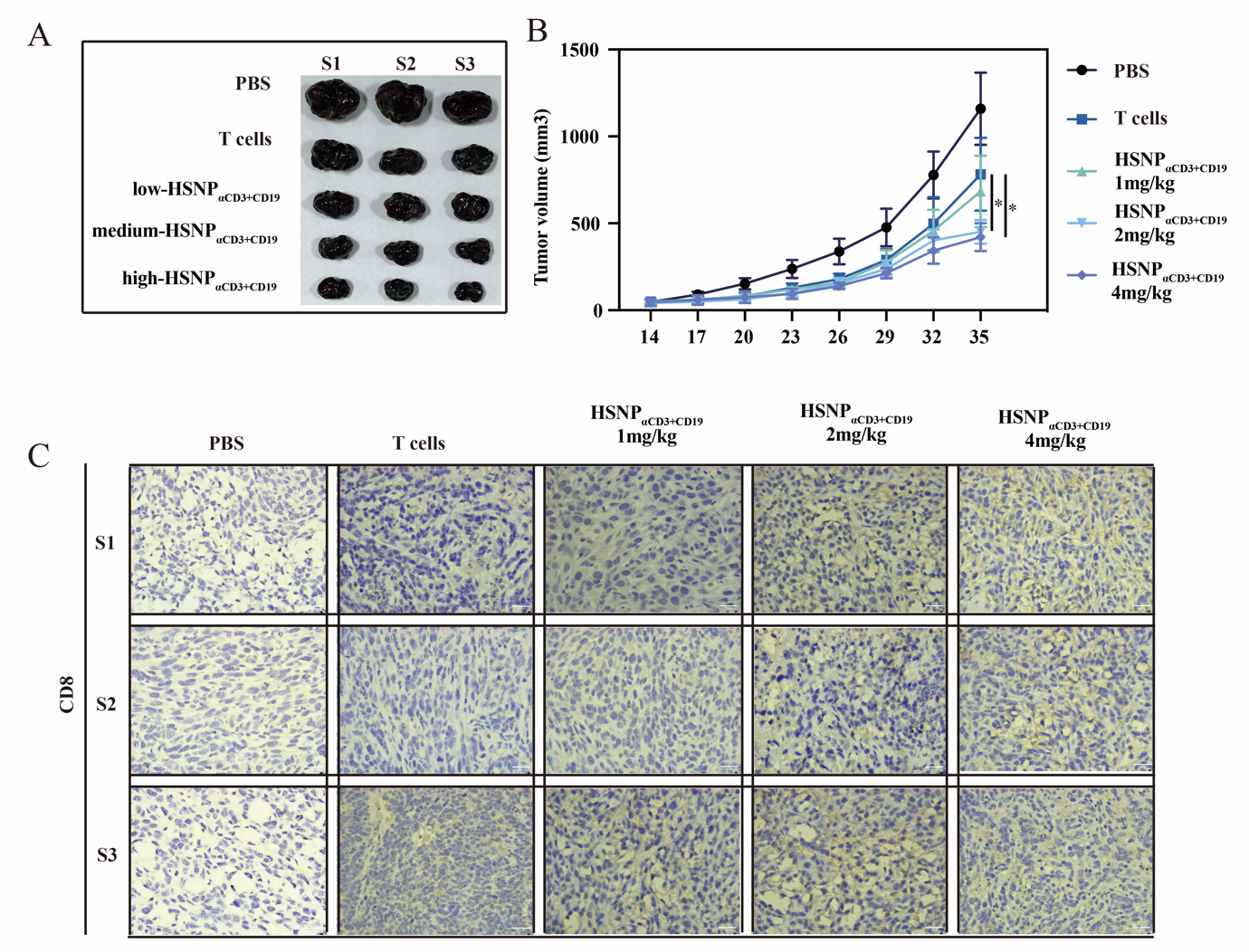


Figure S15. Preliminary in vivo tumor growth inhibition experiments.

After 3 weeks of intervention, the tumors were harvested, weighed, and photographed for documentation. The tumors were then divided into two equal portions: one portion was fixed in 4% paraformaldehyde, and the other portion was frozen and stored at –80 °C. Immunohistochemistry assay was performed to determine the expression and localization of CD8 in the tumor tissue. The model group did not show any significant positive staining. However, positive staining was observed in the remaining four groups, with the range and intensity of positive staining increasing in the order of T cells, low-dose nanoparticles, medium-dose nanoparticles, and high-dose nanoparticles. Statistical differences are analysed by using One-way ANOV and statistical significance is indicated as *P ≤ 0.05.
